# Hydrogels modified with IL4 or SDF1 mimicking peptides serve as a supportive environment for human iPSC-derived myoblasts

**DOI:** 10.1186/s13287-026-05192-1

**Published:** 2026-07-26

**Authors:** Zuzanna Michalska, Anna Ostaszewska, Maria Dzierżyńska, Iwona Grabowska, Małgorzata Zimowska, Martyna Fularczyk, Piotr Skorupiński, Wanda Płoska, Aleksandra Romańska, Alicja Pluta, Zbigniew Bartoszewicz, Katarzyna Jańczyk-Ilach, Sylwia Rodziewicz-Motowidło, Maria A. Ciemerych, Edyta Brzoska, Karolina Archacka

**Affiliations:** 1 Department of Cytology, Faculty of Biology, Institute of Developmental Biology and Biomedical Sciences, University of Warsaw, Warsaw, Poland; 2 Department of Biomedical Chemistry, Faculty of Chemistry, University of Gdańsk, Gdańsk, Poland; 3 Department of Internal Diseases and Endocrinology, Medical University of Warsaw, Warsaw, Poland

**Keywords:** Hydrogel, IL4 peptide, SDF1 peptide, Skeletal muscle, iPSCs, Myoblasts

## Abstract

**Background:**

Skeletal muscles are characterised by substantial regenerative potential, which may be insufficient in case of, e.g., extensive muscle damage, changes accompanying muscle diseases or ageing. For this reason, different ideas are being tested to develop efficient and safe approaches to treat such muscles. Among them, strategies based on biomaterials and stem cells or their derivatives play an important role.

**Methods:**

In the current study, we tested RADA16-I-based hydrogels, modified with custom-designed IL4 or SDF1 mimicking peptides, as a tool that allows development, maintenance or even an increase of myogenic differentiation potential of human myoblasts derived from induced pluripotent stem cells (iPSCs), which are considered a source of cells in therapies for skeletal muscles. Since the release of abovementioned peptides from hydrogels requires activity of metalloproteinases (MMPs) we first assessed the expression of MMPs in iPSC-derived myoblasts cultured on hydrogels. Then we determined the influence of hydrogels on proliferation, apoptosis, and differentiation of iPSC-derived myoblasts as well as their transcriptome and secretome.

**Results:**

We found that the hydrogels designed by us allowed proper proliferation, migration, and myogenic differentiation of hiPSC-derived myoblasts. The latter was even increased in case of hydrogels functionalised with either IL4 or SDF1 peptides. Importantly, none of the hydrogels tested induced neither apoptosis nor unwanted differentiation, i.e. adipogenic, chondrogenic or osteogenic, of hiPSC-derived myoblasts. In response to hydrogel presence, changes in the expression of factors, such as *CXCL8*, *FREM2*,* ADAMTS16*,* CDH9*, *IRS4*,* FIBIN*, or *MLIP* involved in angiogenesis, inflammatory mediation, cell adhesion, neurogenesis, and regulation of myogenic program were observed.

**Conclusions:**

Obtained results indicate that hydrogels functionalised with IL4 or SDF1 peptides serve as a supportive environment for myoblasts derived from hiPSCs and increase features that are beneficial for myogenic/regenerative potential of these cells.

**Supplementary Information:**

The online version contains supplementary material available at https://doi.org/10.1186/s13287-026-05192-1.

## Background

Skeletal muscle regeneration is based on the coordinated cell response to injury or disease. The main players in this process are satellite cells (SCs), i.e., unipotent skeletal muscle stem cells. Changes in skeletal muscle after injury lead to the appearance of many activating factors, which induce proliferation and differentiation of SCs. Among them are cytokines and growth factors released *inter alia* by injured muscle fibers, inflammatory, or endothelial cells [1]. Activated SCs proliferate, differentiate into myoblasts, and fuse to form myotubes that mature into muscle fibers. This process is regulated by myogenic regulatory factors (MRFs), i.e., MYOD, MYF5, MRF4, and myogenin which act as transcription factors involved in the regulation of the expression of multiple muscle-specific proteins. Skeletal muscle reconstruction also requires closely coordinated participation of other cell types present or migrating to this tissue, such as fibroblasts, endothelial cells, nerve cells, immune cells, and fibro/adipogenic progenitors (FAPs).

Interleukin 4 (IL4) and stromal derived factor 1 (SDF1, also called CXCL12) play an important role in both myogenic differentiation and skeletal muscle regeneration. IL4 acts via IL4R and IL13R [2] and is involved in promoting the incorporation of myoblasts into myotubes, allowing their growth as well as formation of muscle fibers. Furthermore, enhanced myoblast migration by IL4 was reported [3, 4]. IL4 treatment affected the expression of β1 and β3 integrins involved in cell migration, as well as factors that participate in the regulation of myogenic differentiation, such as MYOD, myogenin or myomerger [3]. Therefore, IL4 is essential for skeletal muscle regeneration. Our previous studies showed that preconditioning of mouse embryonic stem cells by IL4 improves the competence of such cells for myogenic conversion [5]. On the other hand, SDF1 is a well-known factor promoting stem and progenitor cell migration and mobilisation [6, 7]. SDF1 binds to two receptors: CXCR4 and CXCR7 [7]. SDF1 was shown to increase angiogenesis by provoking VEGF secretion [8]. It also regulates neurogenesis via influence on migration and differentiation of neural precursor cells [9]. Our previous studies showed that SDF1 improved skeletal muscle regeneration by mobilising CXCR4 and CD34 expressing cells [10, 11]. Furthermore, SDF1 enhanced mouse myoblast migration in MMP2 and MMP9 dependent manner [11]. Interestingly, both factors, i.e., IL4 and SDF1 supported regenerative potential of adipose-derived stromal cells and bone marrow-derived stromal cells when overexpressed in such cells or when used for cellular preconditioning [4, 12–14].

As mentioned above, SCs effectively contribute to skeletal muscle reconstruction. However, in some cases such as major injury (e.g., volumetric muscle loss, VML) or disease development, the muscle regeneration is not successful. Myogenic cell transplantation is one of the strategies considered to support skeletal muscle reconstruction [15]. Currently, induced pluripotent stem cells (iPSCs) are considered the most important source of such cells. They have become an easily accessible source of human pluripotent cells since their generation in 2007. The exploration of iPSCs’ potential to produce large numbers of myogenic cells, such as myoblasts, has been highly useful for researchers investigating, e.g., degenerative skeletal muscle diseases, as it could bypass the limitations associated with the use of embryonic stem cells (ESCs) or non-muscle cells with myogenic potential. While ESCs are characterized by great differentiation potential, their use in regenerative medicine is limited by ethical concerns and biological challenges, including their inherently allogenic nature. Non-muscle cells with myogenic potential remain clinically irrelevant because they contribute little to muscle regeneration, have limited *ex vivo* expansion capacity, and face additional hurdles related to their restricted ability to migrate within the tissue and to be effectively delivered to the site of muscle injury. In addition, there are cells with important trophic and immunomodulatory effects, such as MSCs derived from bone marrow or adipose tissue, which could contribute to skeletal muscle regeneration and were previously tested and described in studies performed by us and others e.g [13, 16–18]. Although these cells play important biological role during muscle reconstruction, iPSC-derived myoblasts offer unique advantages for disease modelling, particularly facilitating patient-specific therapeutic approaches, and direct replacement of damaged myogenic cells. For this reason, we chose them as the cellular model in the present study.

Several approaches to induce myogenic differentiation of iPSCs have already been explored. One of them involved direct reprogramming of iPSCs by the overexpression of *PAX7* [19] or genes from the MRF family. Such methods turned out to be very effective, reaching up to 90% efficiency [20, 21], especially by overexpressing *MYOD1*. However, in many of these studies, the use of viral vectors for efficient genetic modification raised concerns about their clinical applicability. Another approach involved the development of protocols which mimic events that occur during muscle tissue development. The methods such as high-throughput screening to identify optimal combinations of small molecules and growth factors for myogenic differentiation, have proven to be highly effective in generating large volume of myogenic cells. Particularly, the single-layer system based on a three-step protocol may serve as a great example of such an approach [22]. At the beginning, hiPSCs were placed at low density on collagen I coated plates and exposed to skeletal muscle medium. After 10 days, immunofluorescence showed that 80% of the cells were PAX3+, 20% PAX7+, and 30%-40% CD56+, confirming their commitment to the myogenic lineage. On day 18, 50%-60% were characterized by the presence of MYOD1 and 20% were DESMIN+, indicating progression to skeletal myoblasts. On day 26, 50%-80% of cells formed multinucleated myotubes expressing myogenin, MF20, dystrophin, and α-actinin. This approach proved that up to 70% of cells expressing skeletal muscle myosin heavy chains can be obtained in less than 30 days, without cell sorting or genetic manipulation. For this reason, this type of protocol has become the current standard procedure for the generation of mature myogenic cells [22].

The exploration of novel therapeutic approaches for dysfunctional skeletal muscles requires not only generation of a substantial number of functional cells but also the maintenance of cell myogenic identity and regenerative potential both *in vitro*, during their propagation and *in vivo*, after their transplantation. It was shown that rigid surfaces could accelerate the loss of valuable cell features for potential skeletal muscle therapy, such as SC stemness, or myoblast differentiation and engraftment potential [23]. Creating an in *vitro* environment similar to the stiffness of naïve skeletal muscle tissue would improve the maintenance of cell myogenic potential or support stem cell directed differentiation towards myogenic lineage [24].

Hydrogels have been considered a valuable biomaterial to create such an *in vitro* environment, suitable for myogenic cells. This is *inter alia* connected with inherent biocompatibility, biodegradability, and highly tailorable mechanical properties of different hydrogel types, thus, enabling support for cell growth and migration. Hydrogel scaffolds can be formed by compounds called self-assembling peptides (SAPs). These hydrogels are characterised by spontaneous gelation upon dissolution in water and could be functionalised by an active signalling sequence attached to their self-assembling motif. A prominent example of such hydrogels is RADA16-I, which consists of periodic repeats of the RADA amino acid sequence. The beneficial effect of RADA hydrogel functionalised with GHK and KGHK motifs was previously shown for effective support of skin wound closure upon biopsy punch wound. GHK was shown to act as a chemoattractant of immune and endothelial cells to the injury site, while KGHK supported tissue vascularization. Both GHK and KGHK were also involved in the regulation of matrix metalloproteinase (MMP) activity, contributing to tissue remodelling [25–27].

Apart from the abovementioned hydrogel, also other may provide a supportive three-dimensional (3D) structure that enhances myogenic cell survival, differentiation, and integration into host tissues after transplantation. Among them, gelatin methacrylate (GelMA), collagen, nanocellulose, fibrin, or alginate have shown the ability to support myogenic cell survival and differentiation, whereas synthetic polymeric scaffolds such as PLGA have mainly been used to improve cell delivery and retention [28]. Combining GelMA with other materials, such as polysaccharides (e.g. alginate) or nanomaterials, improves its mechanical properties and supports myotube formation and alignment, critical for muscle tissue engineering [29]. Collagen-based hydrogels, derived from gelatin, provide structural integrity and mechanical strength, which are essential for muscle tissue repair. These hydrogels mimic the extracellular matrix (ECM), promoting cell adhesion and tissue remodelling [30]. Fibrin promotes differentiation and myotube formation, while alginate induces quiescence [28]. Porous scaffolds, particularly those made from biodegradable materials like PLGA, enhance cell attachment, growth, and differentiation of human myoblasts into myofibers [31]. Importantly, such scaffolds improve the delivery and retention of myogenic cells compared to direct injection into the muscle [32, 33]. For example, implementation of PLGA scaffolds seeded with murine SCs resulted in improved muscle regeneration compared to direct cell injection [32].

In the current study, we used a predetermined RADA16-I sequence, modified with custom-designed IL4 or SDF1 mimicking peptides, which were tested and characterized in our previous study [34], and assessed their influence on proliferation, apoptosis, migration, and differentiation of hiPSC-derived myoblasts (hiPSC-MBs) *in vitro*. Physicochemical characterization of the RADA16-I-based hydrogel is available in [35] while the full physicochemical characterization of the hydrogels investigated in the current study is described in other article (Dzierżyńska et al., *in preparation*). Custom designed RADA16-I-based hydrogel functionalized with SDF1 or IL4 mimicking peptides are mentioned also in our previous *in vitro* study [36] and manuscript accepted for publication (Ostaszewska et al., 2026; https://doi.org/10.1038/s41598-026-49704-8*)* focused on *in vivo* analysis of the effect of hydrogel transplantation on the regeneration of acutely or chronically injured skeletal muscles. We chose this type of hydrogel because of its self-assembling properties, nanofibrous architecture, closely mimicking the organization of the extracellular matrix (ECM), ease of functionalization with bioactive peptide motifs, injectability, and ability to form thin hydrogel coatings that promote cell attachment and migration, while also enabling precise control of biochemical signaling. In addition, the effects of unmodified RADA16-I hydrogels and RADA16-I hydrogels functionalized with the control RGD peptide, a well-established mediator of cell adhesion to the ECM, on hiPSC-MBs were also investigated. Here, the changes in the iPSC-MB transcriptome and secretome, following their culture on thin-hydrogel-coated surfaces were studied. Our main objective was to undertake such a comprehensive assessment of the influence of the above-mentioned hydrogels on different cell features, since these biomaterials could be used not only as preconditioning agents in myogenic cell culture, but also could be transplanted alone or with iPSC-MBs to injured skeletal muscles.

## Methods

### Preparation of functionalized peptide hydrogels

Heated Solid Phase Peptide Synthesis (SPPS) was performed using an automated peptide synthesiser (Activotec, Cambridge, UK) to obtain crude peptides, as previously described [26]. Tentagel R RAM resin was used, with diisopropylcarbodiimide (DIC) and Oxyma Pure as coupling reagents. Fmoc group deprotection was carried out using 25% piperidine in “magic mixture” (DMF, NMP, DCM − 1:1:1 v/v/v), containing 1% Triton X-100 detergent. The synthesis was completed with acetylation using *N*-acetylimidazole. Peptides were cleaved from the resin along with their protecting groups using a cleavage cocktail consisting of trifloroacetic acid (TFA), triisopropylsilane (TIS), phenol, and water (ratio 88:2:5:5 v/v/v/v). The crude peptides were then precipitated in cold diethyl ether, centrifugated, suspended in water, and lyophilised. Every peptide was then purified using reverse-phase high-performance liquid chromatography (HPLC) with a Jupiter Proteo C12 (4 μm, 90 Å) column (Phenomenex, Torrance, CA, USA). Full physicochemical characterisation of the peptides was performed [34].

### Gelation process

Purified peptides were sterilized in solid form using a UV lamp (λ = 254 nm). Reconstitution was carried out under a laminar flow cabinet to achieve a final concentration of 1% (w/v). The samples were vortexed and sonicated until a clear solution was observed. Hydrogel formation was induced by incubating the peptide solutions at 37°C in a thermoblock overnight. The pH was measured immediately after peptide dissolution using indicator strips, and adjustment was performed only when the pH values deviated from the optimal range for peptide self-assembly, it was adjusted to approximately pH 5 to facilitate the assembly process. The functionalized peptide (R1-RGD, R1-SDF1-X, R4-RGD or R4-IL4-Y) was used at a final concentration of 5 mg/ml and mixed in equal amounts of RADA16-I (final concentration of 5 mg/ml). Thus, the final hydrogels were mixed with unmodified RADA16-I (R1) (1:1 v/v). Such a procedure resulted in a material of peptide concentration of 10 mg/ml (1% w/v, equivalent to 10 g/L).

### Cell lines and cell culture

Two commercially available human induced pluripotent stem cell lines were used: hiPSC-DMD-EGFP (MONO-ALLELIC mEGFP-TAGGED DMD, Allen Institute for Cell Science), and hiPSC-LMNβ1-EGFP (MONO-ALLELIC mEGFP-TAGGED LMNβ1, Allen Institute for Cell Science). Cells were characterized by either *DMD* or *LMN*β*1* being fluorescently tagged. Undifferentiated hiPSC colonies were cultured on vitronectin-coated plates in Essential 8 Medium (Thermo Fisher). Truncated human recombinant vitronectin (A14700, Gibco) was used to coat cell culture plates according to manufacturer’s instruction. Briefly, vitronectin was suspended in sterile PBS to reach 5 µg/ml concentration, added to 6-well plate (Falcon) and incubated at room temperature for 1 h. Next, vitronectin solution was discarded and cells were seeded directly onto prepared wells. hiPSC myogenic differentiation was performed using commercially available differentiation medium (SKM-KITM, Genea Biocells). Undifferentiated hiPSCs were dissociated using Accutase (Thermo Fisher) and seeded onto collagen I coated plates (Corning) at the density of 5.000 cells/cm^2^. The first stage of differentiation was performed using Skeletal Muscle Induction Medium (SKM01, Genea Biocells) and aimed to generate myogenic precursors, expressing *PAX7*. After 6–10 days in culture, the medium was switched to Skeletal Myoblast Medium (SKM02, Genea Biocells) to induce myogenic precursor cell conversion towards myoblasts for an additional 6–8 days. Once hiPSC-derived myoblasts (hiPSC-MBs) were generated, the cells were subjected to *in vitro* experiments.

### Hydrogel coating

To evaluate hydrogel impact on hiPSC-MBs, the cells were seeded onto previously hydrogel-coated 24 well plates (Falcon). We have evaluated the impact of four peptide-functionalised hydrogels: R1-SDF1-X and R4-IL4-Y, as well as R1-RGD and R4-RGD, modified with a control RGD peptide [36]. As a control hydrogel, an unfunctionalised RADA16-I hydrogel was used (described hereinafter as R1). Each well was covered with a thin (~ 10 µl) hydrogel layer and incubated at 37°C for 4 h to polymerise. The hydrogel coated wells were covered with SKM02 and incubated overnight at 37°C. The following day, the hydrogel layer was rinsed twice with SKM02 to equilibrate pH and immediately after used for hiPSC-MB culture.

### Apoptosis assay

Apoptosis of hiPSC-MBs was quantified using the Annexin V APC (R37176, Invitrogen) according to the manufacturer’s instructions. Briefly, after 72 h of culture in 24-well plates (Falcon) coated with different hydrogels or on collagen I coated plates (Corning), serving as control, in SKM02 medium, the cells were trypsinised and collected into FACS tubes. Annexin V-APC (Thermo Fisher) was added to the cell suspensions, incubated for 10 min in darkness at room temperature, and subjected to flow cytometry analysis (CytoFLEX, Beckman Coulter) with CytExpert (Beckman Coulter) software. Unlabelled cells (negative control) were included in each experiment.

### Proliferation assay

To assess the proliferation of the hiPSC-MBs, BioTracker™ 488 Green CFSE Cell Proliferation Kit (SCT110, Sigma Aldrich) was used according to the manufacturer’s instructions. Briefly, hiPSC-MBs were seeded at a density of 5.000 cells/cm^2^ on 24-well plates (Falcon) coated with different hydrogels or collagen I (control) and maintained in SKM02 for 24 h. The medium was removed and cells were incubated for 15 min at 37°C with 0.5 µM carboxyfluorescein succinimidyl ester (CFSE, Merck Millipore) to allow dye uptake. The staining solution was replaced with a pre-warmed SKM02 and incubated for 5 min to allow hydrolysation of the free dye. After that time, the medium was replaced with fresh SKM02 and incubated for an additional 30 min to allow CFSE to react with intracellular proteins. Then, fresh SKM02 was replaced again and hiPSC-MBs were incubated for 48 h to allow the cells to divide. All incubation steps were performed at 37°C, and cells were protected from light by wrapping the plate with aluminium foil. After 2 days, cells were collected and subjected to flow cytometry analysis (CytoFLEX, Beckman Coulter) with CytExpert software (Beckman Coulter). Unlabelled cells (negative control) and cells analysed directly after labelling with CFSE (positive control) were included in each experiment.

### Migration assay

To assess the impact of hydrogels on hiPSC-MB migration, a QCMTM 24-well colorimetric cell migration assay (Merck Millipore) was used. The assay was performed in a dish, containing inserts with 8 μm pores (Merck Millipore). The bottom wells were covered with tested hydrogels and incubated at 37°C for 4 h to allow hydrogel polymerisation. Next, SKM02 medium was added to the hydrogel-coated plates which were incubated at 37°C for 24 h. Simultaneously, hiPSC-MBs were seeded on the inserts at the density of 14 000 cells/cm^2^, to allow cell attachment to the polycarbonate membrane. The wells covered with hydrogels were then rinsed twice with SKM02 to allow pH stabilisation and inserts with cells were transferred to the wells with hydrogels. Both the wells and the inserts were filled with SKM02 medium. Cells were maintained in culture for 18 h. Then the medium was discharged, the inserts transferred to a new well containing 400 µl of cell stain (90144, Sigma Aldrich) and incubated for 20 min. The inserts were rinsed a few times with distilled water, and the cells from the top of the insert (i.e., those that did not migrate) were removed using cotton swabs. The inserts were allowed to air dry, transferred to a well containing 200 µl of extraction buffer (40145, Sigma Aldrich) and incubated for 15 min. After that time, the inserts were removed from the well and 100 µl of each cell extract was transferred to a 96-well plate (351172, Thermo Fisher). Optical density was measured at 560 nm in the Multiskan SkyHigh spectrophotometer (A51119600C, Thermo Scientific).

### Scratch wound healing assay

Cell migration was also tested using a scratch wound healing assay. These experiments were carried out using Culture Insert 2 24-well plates (80242, Ibidi). Cells were seeded at the density of 10.000 cells/cm^2^ in each inner well and maintained in culture in SKM02 until confluence. The culture inserts were removed, creating a cell-free gap in each well. The wells were further filled with SKM02 medium and incubated at 37°C for 18 h to allow cell migration. After that time, cells were fixed with 3% PFA in PBS and stained with Giemsa and May-Grünwald dyes (109204, Sigma Aldrich; 101424, Sigma Aldrich). The wound area was photographed with a Nikon eclipse TE200 microscope and a Nikon Digital Sight DS-U2 camera. The number of cells that migrated to the gap area was counted using ImageJ (Fiji).

### hiPSC-MB differentiation

hiPSC-MBs were seeded in hydrogel-coated plates and maintained in SKM02 for 3 days, followed by 6 days in myotube induction medium (SKM03, Genea Biocells). Next, cells were fixed with 3% PFA in PBS and stained with Giemsa and May-Grünwald dyes to visualise nuclei and cytoplasm. The samples were next photographed using the Nikon eclipse TE200 microscope and Nikon Digital Sight DS-U2 camera. The captured photos were analysed using Image J (NIS-Elements F 2.30). The fusion index was calculated as the number of nuclei inside the myotubes divided by the total number of nuclei present in the photo.

### Adipogenesis, chondrogenesis, and osteogenesis assay

hiPSC-MBs’ adipogenic, chondrogenic, and osteogenic potential was evaluated following the culture in differentiation inducing media: for adipogenesis, AdipoMAX (SCM122, Sigma Aldrich), for chondrogenesis, ChondroMAX (SCM123, Sigma Aldrich), and for osteogenesis - OsteoMAX (SCM121, Sigma Aldrich). Initially, the cells were seeded on hydrogel coated plates and maintained in SKM02 until confluence, and next exposed to the above-mentioned differentiation media. Adipogenesis was analysed within 14 days, chondrogenesis after 21 days, and osteogenesis within 7 days after differentiation.

### Oil red O staining

hiPSC-MBs were fixed with 3% PFA in PBS for 15 min, followed by cell rinsing with tap water. The cells were rinsed with 70% isopropanol (563935, Sigma Aldrich) and stained with Oil Red O solution (O1391, Sigma Aldrich). Before use, the Oil Red O solution was prepared by mixing 120 ml of Oil Red O with 80 mL of distilled water. The solution was incubated for 10 min and filtered through a syringe filter. Cells were incubated in cell dye at room temperature for 30 min. The cells were rinsed three times with distilled water and photographed with a Nikon eclipse TE200 microscope and a Nikon Digital Sight DS-U2 camera.

### Alcian blue staining

hiPSC-MBs were fixed using 3% PFA in PBS for 15 min. The cells were rinsed twice with PBS, followed by a 3 min incubation in 3% acetic acid. The cells were incubated with Alcian Blue Staining Solution (TMS-010-C, Sigma Aldrich) at 37°C and gentle shaking for 30 min. After rinsing with 3% acetic acid, cells were covered with tap water for 10 min. Finally, the cells were rinsed with distilled water and photographed using a Nikon eclipse TE200 microscope and a Nikon Digital Sight DS-U2 camera.

### Alizarin red staining

Fixed hiPSC-MBs (3% PFA in PBS, 15 min) were rinsed three times with distilled water and covered with Alizarin Red S solution (TMS-008, Sigma-Aldrich) at room temperature with gentle shaking for 30 min. The cells were rinsed three times with distilled water and photographed with a Nikon eclipse TE200 microscope and a Nikon Digital Sight DS-U2 camera.

### RT-qPCR

Total RNA was isolated from cell pellets using High Pure RNA Isolation Kit (11828665001, Roche), according to the manufacturer’s instructions, followed by determination of RNA concentration and contamination using NanoDrop (Thermo Scientific). Next, reverse transcription was performed using 0.5 µg RNA for each reaction and the RevertAid First Strand cDNA Synthesis Kit (K1622, Thermo Fisher), according to the manufacturer’s instructions. Quantitative polymerase chain reaction (qPCR) was performed using TaqMan Gene Expression Master Mix (4369016, Thermo Fisher) and TaqMan probes, according to the previously published protocol [13, 18]. For each RT-qPCR reaction, 25 ng of cDNA was used. *GAPDH* served as a reference gene. The analysis was performed with LightCycler^®^ 96 Software 1.1 (Roche). Data are shown as ΔCT or RQ (2^−ΔΔCT^) values. All TaqMan probes used (Thermo Fisher) are listed in Supplementary Table 1.

### Next generation sequencing

Total RNA (1000 ng) from iPSC-MBs cultured either on collagen I, non-functionalised R1 or peptide functionalised R1-SDF1-X or R4-IL4-Y hydrogels, was isolated using mirVana™ miRNA Isolation Kit (AM1561, Thermo Fisher), according to the manufacturer’s instructions. RNA purification and NGS analysis was outsourced to the external facility (Genomics Core Facility, Centre of New Technologies, University of Warsaw, Poland). RNA samples were purified using the RNA Clean & Concentrator-5 kit (Zymo Research) and analysed with Agilent Tape Station 2200 using High Sensitivity RNA ScreenTape (Agilent). The samples were subjected to ribodepletion and library construction using 500 ng of RNA and the KAPA RNA HyperPrep Kit with RiboErase (KAPA Biosystems) according to the manufacturer’s protocols. The KAPA_U DI_Indexes (96 Indexes, 96 Samples; KAPA Biosystems) were used. Next, the samples were fragmented: 94°C, 5 min, and 8 enrichment cycles were performed. The resulting libraries were subjected to fragment length control using Agilent Tape Station 2200 and High Sensitivity D1000 Reagents (Agilent) according to the manufacturer’s protocol. Sequencing was performed on an Illumina NovaSeq 6000 instrument using NovaSeq 6000 S1 Reagent Kit v1.5 (200 cycles) reagents (Illumina), in a pair-end read mode 2 × 100 cycles using the standard procedure recommended by the manufacturer with the 0.5% addition of the Phix control library (Illumina). The raw sequences were trimmed according to quality using Trimmomatic (version 0.39 [37]), Trimmed sequences were mapped to the human reference genome provided by ENSEMBL (version grch38_snp_tran) using Hisat2 [38] with default parameters. Optical duplicates were removed using the MarkDuplicates tool from the GATK package [39] (version 4.2.3.0). The mapped reads were associated with transcripts from the grch38 database [40](Ensembl, version 106) using HTSeq-count [41](version 2.0.1). Differentially expressed genes were selected using the DESeq2 package [42](version 1.38.3). Over-representation of the terms of Gene Ontology [43] and Kyoto Encyclopedia of Genes and Genomes (KEGG) [44] categories among the top 5% of genes (according to the p-value) were assessed with clusterprofiler package [45]. The raw sequence data is available in repository:

https://danebadawcze.uw.edu.pl/dataset.xhtml? persistentId=doi:https://doi.org/10.58132/XYKGK1.

### Growth factor analysis (luminex)

Analysis was performed as previously described [46]. The concentration of BMP2, IL4, IL8 (CXCL8), SDF1, and VEGF-A was measured using the Human Premixed Multi-Analyte kit number LXSAH (R&D Systems) on Bio-Plex MAGPIX Multiplex Analyzer (Luminex Corp.). A Bio-Plex Manager 6.1 (Bio-Rad Lab Inc.) was used for the generation of the 5-log standard curve, the fitting of the curve and calculation of concentrations. Results from all experiments were normalised against control cells, i.e., those cultured on collagen I.

### Immunocytochemistry

hiPSC-MBs were cultured on 1% gelatine until reaching 80–90% confluence. The cells were fixed with 3% PFA, washed in PBS, and proceeded for immunodetection of MMPs, necessary for the release of the SDF1 or IL4 peptide from functionalised hydrogels. As we described previously, SDF-1 or IL-4 was linked to RADA16-I peptide through MMP-degradable sequence [34]. We focused on MMPs (described below) that could potentially cleave the enzymatic linkers within R1-RGD, R1-SDF1-X, R4-RGD and R4-IL4-Y structures. After cell membrane permeabilization by 1 min incubation in 0.05% Triton X-100 in PBS, free aldehyde groups were blocked with 0.25% glycine in PBS and then nonspecific antigen binding sites were blocked by 1 h incubation in 3% bovine serum albumin (BSA) in PBS. Then, overnight incubation with primary antibody, at 4°C, was performed, followed by three washes with PBS. The following primary antibodies were used: MMP2 (ab97779, Abcam), MMP3 (ab52915, Abcam), MMP7 (ab207299, Abcam), MMP9 (ab76003, Abcam), MMP13 (ab39012, Abcam), MMP14 (ab51074, Abcam). Next, 2 h incubation with secondary antibody (goat anti-rabbit; ab150077, Abcam) diluted in PBS was performed and cells were washed three times with PBS. Cell nuclei were stained with Hoechst 33,342 (B-2261, Sigma Aldrich), diluted 1:1000 in PBS. Eventually, cells were mounted on slides (S3023, Dako) and analysed using an Axio Observer Z1 LSM 700 scanning confocal microscope equipped with ZEN software.

### Zymography in situ

To detect the activity of MMP2 and MMP9, hiPSC-MBs were cultured for 3 days on gelatine-coated glass slides placed in a 6-well plate. 30 µl of gelatine-fluorescein conjugate (100 µg/mL; Thermo Fisher) was added to each well and incubated at 37°C for 45 min. Next, the cells were fixed with 3% PFA in PBS for 15 min. Nuclei were stained with Hoechst 33342 (B-2261, Sigma Aldrich), diluted 1:1000 in PBS. Finally, the specimens were closed with mounting medium (S3023, Dako). Cell images were taken using an Axio Observer Z1 LSM 700 scanning confocal microscope equipped with ZEN software.

### Zymography in gel

Cell-conditioned media were collected after 3 days of hiPSC-MB culture and the enzymatic activity of MMP7 (substrate casein; Sigma Aldrich; concentration 0.1% in gel) or MMP2 and MMP9 (substrate gelatine; Sigma Aldrich, concentration 1% in gel) was tested. To condense cell media, sepharose conjugate (GE Healthcare) was prepared by mixing it with 20% ethanol, rinsing with distilled water and suspending in incubation buffer containing 50 mM Tris-HCl, 150 mM NaCl, 5mM CaCl_2_, and 0.05% Brij 35 (Sigma Aldrich). Next, 0.5 ml of cell culture media was mixed with 50 µL of sepharose conjugate. The samples were incubated at 4°C with constant shaking for 90 min. Next, the samples were centrifuged (5000 RPM, 5 min) and the supernatant was discarded. The remaining pellet was suspended in buffer B containing 50 mM Tris-HCL, 150 mM NaCl, 5 mM CaCl_2_, 0.05% Brij 35, pH = 7.6 and centrifuged twice in the same conditions. The pellet was suspended in the incubation buffer supplemented with 10% DMSO and incubated for 30 min at 4°C with constant shaking. After that, the samples were centrifuged for 5 min at 5000 RPM and the supernatant was collected. 50 µl of each sample was then mixed with a non-reducing sample buffer containing 62.5 mM Tris-HCl, 10% glycerol, 2% SDS, 0.05 bromophenol blue, and loaded onto 10% SDS-polyacrylamide gels containing 0.1% gelatine (for MMP2 and MMP9) or 0.1% casein (for MMP7). After electrophoretic separation, the gels were washed in 2.5% Triton X-100 in distilled water (2 × 15 min at 37°C with shaking) to discard remaining SDS, followed by 48 h of incubation in buffer containing 50 mM Tris-HCl, 5 mM CaCl_2_, and 200 mM NaCl. After that, the gels were stained with Coomassie Brilliant Blue (1610436, Bio-Rad). The activity of the tested metalloproteinases was visualised by white bands indicating substrate degradation by either MMP2, -7, or -9. The gels were scanned and analysed using GelDoc Quantity One software (Bio-Rad).

### Statistical analysis

At least three independent experiments were performed for each analysis. Comparisons between experimental groups were performed using ANOVA, Kruskall-Wallis or Brown-Forsythe and Welch ANOVA (depending on meeting the normality criterion or SD equality). Correction for multiple comparisons was performed using Tuckey’s, Dunn’s, or Dunett’s T3 post hoc tests, respectively. Error bars represent the mean ± standard deviation. Statistical significance was shown in accordance with GraphPad style, where * *p* ≤ 0.05, ** *p* ≤ 0.01, *** *p* ≤ 0.001, and **** *p* ≤ 0.0001. Statistical analysis was performed using GraphPad Prism 10 software. Figures: Figures were assembled using Adobe Illustrator.

## Results

### Evaluation of myogenic differentiation progression in human iPSC lines

In the current study 2 lines of hiPSCs were used, that is, hiPSC-DMD and hiPSC-LMNβ1. Both cell lines were induced to undergo myogenic differentiation and analysed at 4 different stages, i.e., as (1) undifferentiated cells (2), myoprecursors (3), myoblasts (4), myotubes (Supplementary Fig. 1A) to verify whether their differentiation was accompanied by proper changes in myogenic marker expression. We determined the expression level of *PAX7* mRNA which is characteristic for SCs as well as cells at the early stage of myogenic differentiation and *MYOD* mRNA encoding MRF that is expressed in cells at further and more advanced stages of myogenic differentiation. Furthermore, we evaluated the expression level of mRNAs that encode 3 different types of myosin heavy chains (*MYHC*), i.e., *MYHC2*,* MYHC3*, expressed in developing muscles, and *MYHC7*, which appear in cells in more advanced steps of differentiation [47].

RT-qPCR analysis confirmed that in both cell lines, the undifferentiated hiPSCs were characterised by initial lower levels of all myogenic markers compared to myoprecursors (Supplementary Fig. 1). The expression of all analysed markers was then significantly upregulated in cells that underwent myogenic differentiation (Supplementary Fig. 1C). The highest level of *PAX7* expression was found in myoprecursors from both cell lines and was significantly elevated compared to both undifferentiated hiPSCs and more differentiated myogenic derivatives, i.e., myoblasts and myotubes (Supplementary Fig. 1C). The myotubes, generated from both cell lines, were in turn characterised by the highest level of *MYOD* expression, and this difference was statistically significant compared to all other samples (i.e., undifferentiated cells, myoprecursors, myoblasts; Supplementary Fig. 1C). The level of mRNAs encoding all the analysed *MYHCs* (i.e., *MYHC2*,* MYHC3*,* MYHC7*) was also significantly elevated in myotubes in both cell lines, and also in myoprecursors of hiPSC-LMNβ1 (Supplementary Fig. 1C). The obtained results confirmed that hiPSCs were successfully induced to undergo myogenic differentiation, what was accompanied by expected changes in myogenic marker expression.

### Expression and activity of metalloproteinases in hiPSC-derived myoblasts cultured on hydrogels modified with IL4 or SDF1 peptides

In the next experiments, we investigated the expression and activity of selected types of metalloproteinases (MMPs) in myoblasts derived from each hiPSC cell line (hereafter described as MB-DMD and MB-LMNβ1, respectively). MB-DMD and MB-LMNβ1 were cultured on different types of hydrogels (R1, R1-RGD, R1-SDF1-X, R4-RGD, R4-IL4-Y) or on collagen I, which served as a control. The analysis of MMPs was a pivotal stage of this research, as our hydrogels were modified with IL4 or SDF1 mimicking peptides by linking them to the hydrogel with a sequence recognised by them, i.e. PAGL or VLGL [48]. These enzymes are crucial for releasing IL4 and SDF1 mimicking peptides from hydrogels. We focused on MMP2, MMP3, MMP9, MMP13, and MMP14 as these MMPs could potentially cleave R4-RGD and R4-IL4-Y hydrogels containing the PAGL sequence, as well as on MMP7 which could potentially cleave R1-RGD and R1-SDF1-X ones, containing VLGL sequence.

RT-qPCR analysis revealed that the initial level of MMP expression was similar in both types of hiPSC-derived myoblasts cultured on collagen I (described as cells at day 0, expression level shown in Supplementary Fig. 2A). According to the mean ΔCT values, the highest expression level was found for *MMP2* while other analysed MMPs were expressed at medium (*MMP9*,* MMP14*) or low (*MMP3*,* MMP7*,* MMP13;* Supplementary Fig. 2A). Next, we cultured MB-DMD and MB-LMNβ1 on different hydrogels or on collagen I for three days and assessed MMP expression. The primary aim of this analysis was to determine whether culture on RADA16-I-based hydrogels alters MMP expression compared to the standard collagen I substrate. Both MB-DMD and MB-LMNβ1 cultured on the R4-IL4-Y hydrogel were characterised by significantly higher expression of *MMP2*, *MMP9*, and *MMP14* than control myoblasts (Supplementary Fig. 2B, 2C). *MMP2* was also elevated in MB-DMD and MB-LMNβ1 cultured on the R4-RGD hydrogel (Supplementary Fig. 2B, 2C). All indicated MMPs (*MMP2*,* MMP9*,* MMP14*) could potentially recognise and cleave linking sequence (PAGL) in the R4-RGD and R4-IL4-Y hydrogels, releasing the attached peptides; thus, their elevated expression in myoblasts cultured on such hydrogels was beneficial. *MMP9* and *MMP14* were also elevated, but only in MB-LMNβ1 cultured on the R4-RGD hydrogel (Supplementary Fig. 2B, 2C). Other significant changes in MMP expression between myoblasts cultured on different hydrogels and those cultured on collagen I are shown in Supplementary Fig. 2B and summarised in Supplementary Fig. 2C.

**Fig. 1:**
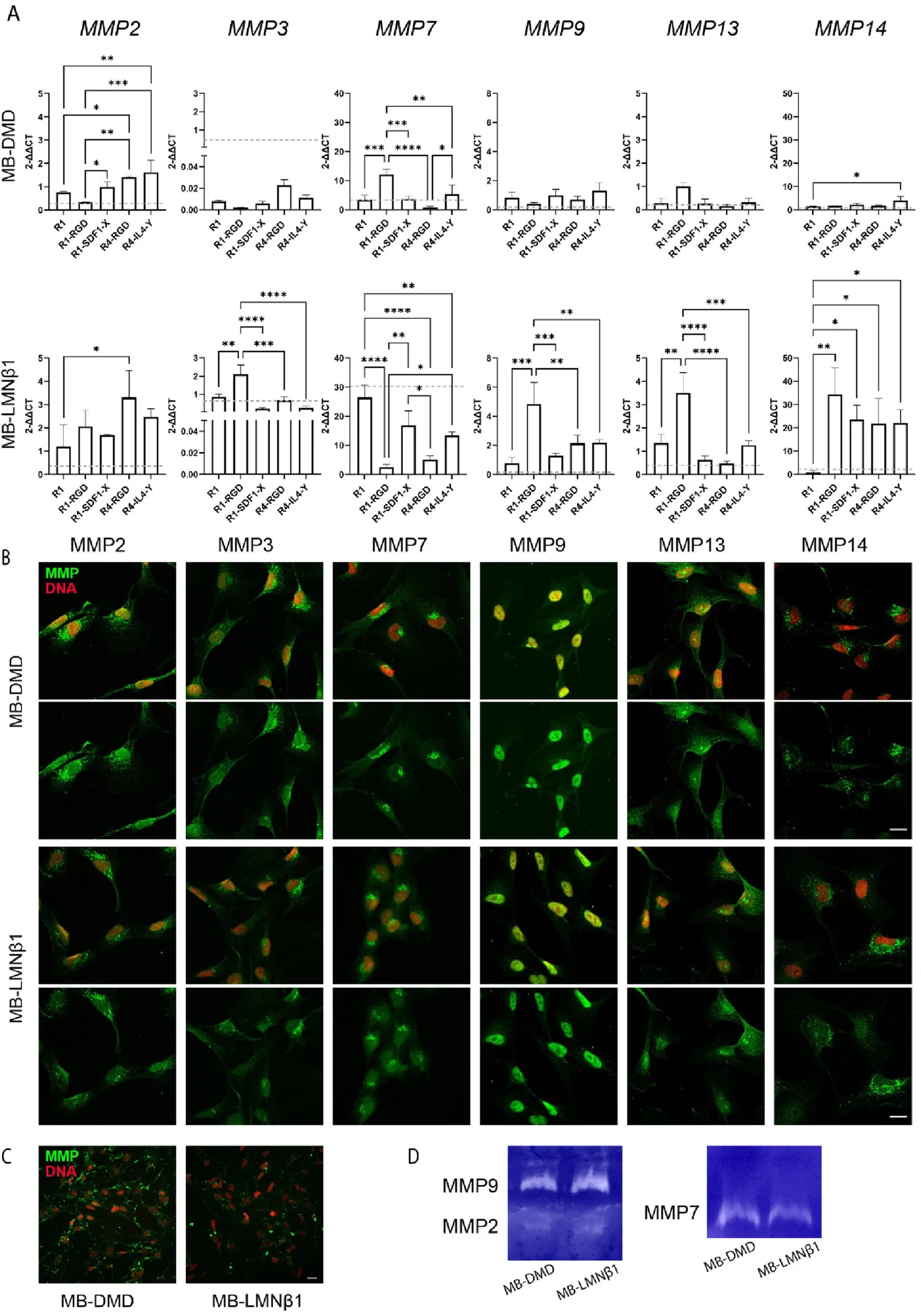
Expression and activity of selected MMPs in hiPSC-derived myoblasts cultured on different hydrogels. **A**. Expression of *MMP2*, *MMP3*, *MMP7*, *MMP9*, *MMP13*, *MMP14* in MB-DMD and MB-LMNβ1 cultured for 3 days in SKM02 on different hydrogels (R1, R1-RGD, R1-SDF1-X, R4-RGD, R4-IL4-Y). The mean RQ (2^−ΔΔCT^) values with standard deviation of at least 3 experiments are shown. The myoblasts on day 0 served as a reference sample. *p ≤ 0.05, ** *p* ≤ 0.01, *** *p* ≤ 0.001, **** *p* ≤ 0.0001. The grey line indicates the level of expression in control cells cultured on collagen I. **B**. Immunolocalization of indicated MMP proteins in MB-DMD and MB-LMNβ1 cultured on gelatin-coated cover slides. Nuclei are visualised in red, MMPs in green; Scale bar − 40 μm; **C**. *In situ* zymography showing gelatinolytic activity in *in vitro* cultured MB-DMD and MB-LMNβ1 on day 3. The nuclei are visualised in red, and gelatinolytic activity is visualised in green; **D**. *Ingel* zymography detecting the activity of MMP2, MMP7, and MMP9 in medium collected after 3 days of MB-DMD and MB-LMNβ1 culture on collagen I

The expression of *MMP2* in MB-DMD and MB-LMNβ1 was significantly higher when cells were cultured on R4-RGD hydrogel, compared to control cells cultured on collagen I (Supplementary Fig. 2B, 2 C), but also to myoblasts propagated on the control R1 hydrogel (Fig. 1A). In the case of MB-DMD, it was also higher compared to cells cultured on R1-RGD (Fig. 1A). Additionally, MB-DMD cultured on the R4-IL4-Y hydrogel expressed significantly more *MMP2* than cells cultured on the R1 or R1-RGD (Fig. 1A). Therefore, *MMP2*, which could potentially cleave the linker peptide in the R4-RGD and R4-IL4-Y hydrogels, was significantly elevated in myoblasts cultured on these hydrogel types, compared to cells cultured on other hydrogel variants (Fig. 1A). In case of other MMPs that can cleave the linker peptide in R4-RGD and R4-IL4-Y hydrogels, that is, *MMP3*, *MMP9*, and *MMP13*, no significant differences were found for MB-DMD cultured on different hydrogel coatings (Fig. 1A). In MB-LMNβ1 significantly higher expression of all indicated MMPs (*MMP3*, *MMP9* and *MMP13*) was noticed in cells cultured on R1-RGD hydrogel compared to other experimental groups (Fig. 1A). The expression of *MMP14*, the last analysed MMP potentially cutting linker peptide of the R4-RGD and R4-IL4-Y hydrogels, was significantly higher in MB-DMD cultured on the R4-IL4-Y hydrogel (Fig. 1A). Furthermore, the expression of *MMP7* that could potentially cleave linker peptide in the R1-RGD and R1-SDF1-X hydrogels was significantly enhanced in MB-DMD cultured on the R1-RGD hydrogel in comparison to other experimental groups while in the case of MB-LMNβ1 it was elevated in cells cultured on the R1, R4-IL4-Y, and importantly, on R1-SDF1-X hydrogel (Fig. 1A). We showed that mRNAs encoding all analysed MMPs were detectable in myoblasts derived from hiPSCs and cultured on all types of hydrogels tested, indicating that such cells could potentially be able to release IL4 and SDF1 mimicking peptides linked to functionalised hydrogels.

To verify whether MMP proteins are present and active in hiPSC-derived myoblasts, we performed immunocytochemistry and zymography *in situ* and *in gel*. Although RT-qPCR results cannot be directly transferred to ICC or zymography analysis, they served for us as an indicator of expression trend in the analyzed cells. Since MMP expression level was usually higher in cells cultured on hydrogels, compared to those cultured on collagen I (Supplementary Fig. 2), we performed ICC and zymography analysis using cells cultured on gelatin/collagen I. All analysed MMPs were successfully detected by immunocytochemistry in both MB-DMD and MB-LMNβ1 (Fig. 1B). MMP2, MMP7, and MMP9 were also evaluated using zymography in which we confirmed the activity of the analysed MMPs (Fig. 1C and D). Taken together, all MMPs required for peptide release from the hydrogel backbone were detected at both the transcript and protein level in myoblasts obtained from both hiPSC lines. The presence of custom-designed hydrogels modified with IL4 and SDF1 mimicking peptides had a noticeable impact on the expression of some analysed MMPs in hiPSC-derived myoblasts, as shown by comparison with controls (collagen I and R1).

### Proliferation, apoptosis, and myogenic differentiation of human iPSC-derived myoblasts cultured on custom-designed hydrogels modified with IL4 or SDF1 peptides

**Fig. 2:**
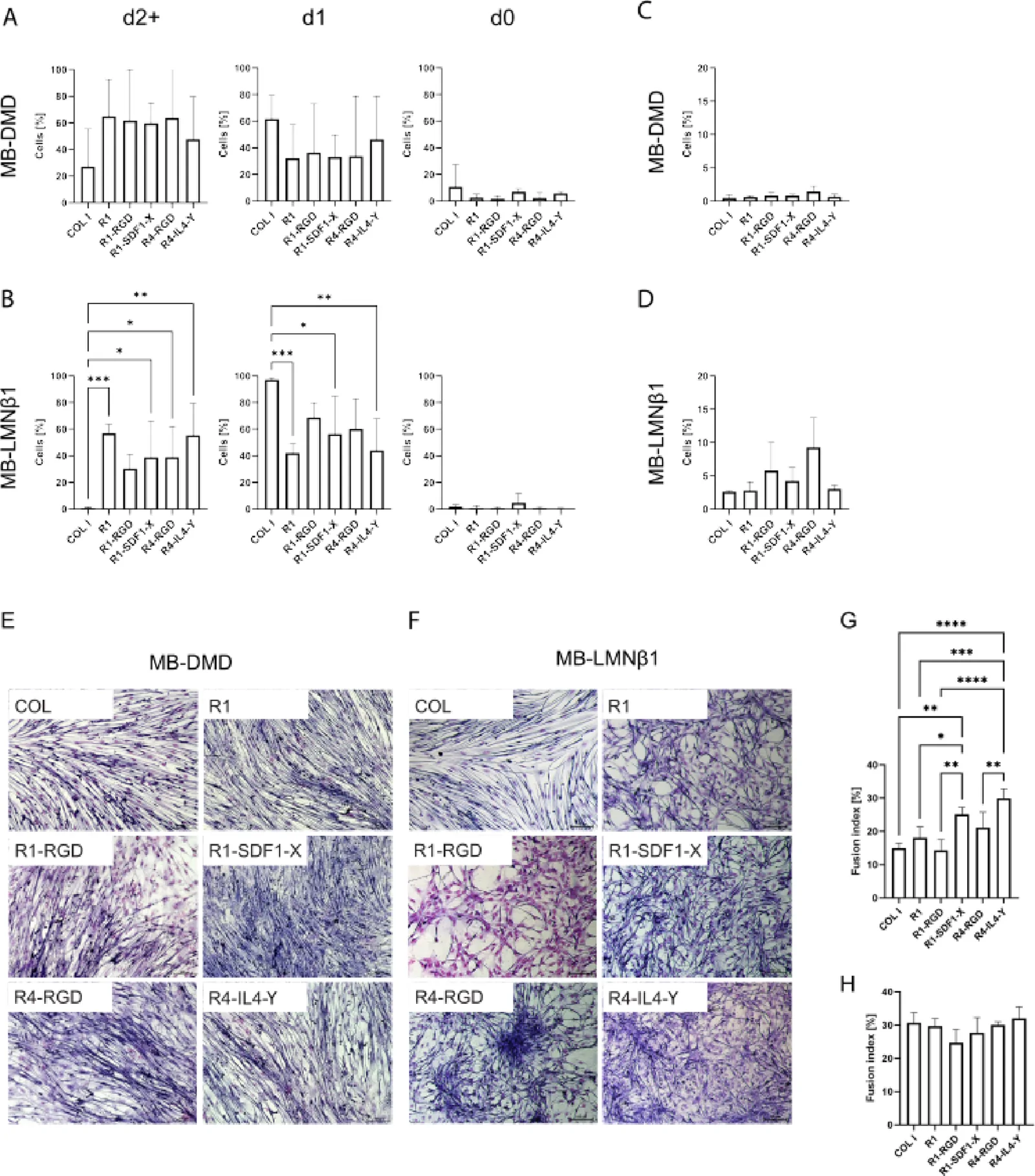
Proliferation, apoptosis, and myogenic differentiation of hiPSC-derived myoblasts cultured on different hydrogels. **A**. The percentage of MB-DMD that underwent two or more cellular divisions (d2+), one (d1), or none (d0) during 48 h culture on the indicated hydrogel types (R1, R1-RGD, R1-SDF1-X, R4-RGD, R4-IL4-Y), or on collagen I (COL I), i.e. in control variant. Data are shown as mean ± SD; **B**. The percentage of MB-LMNβ1 that underwent two or more (d2+), one (d1), or none (d0) cellular divisions during 48 h culture on indicated hydrogels or on collagen I. Data are shown as mean ± SD; *p ≤ 0.05, **p ≤ 0.01, ***p ≤ 0.001; **C**. The percentage of apoptotic cells after 72 h of MB-DMD culture on indicated hydrogels or collagen I; **D**. The percentage of apoptotic cells after 72 h of MB-LMNβ1 culture on the indicated surfaces; **E**. Representative photos of myotubes formed during MB-DMD culture on indicated surfaces, stained with Giemsa-May Grünwald. Scale bar − 100 μm; **F**. Representative photos of myotubes formed during MB-LMNβ1 culture on the indicated surfaces, stained with Giemsa-May Grünwald. Scale bar − 100 μm; **G**. Fusion index of MB-DMD cultured on the indicated surfaces; **D**. Fusion index of MB-LMNβ1 cultured on indicated surfaces

The impact of custom-designed hydrogels modified with IL4 or SDF1 mimicking peptides on proliferation, apoptosis, and myogenic differentiation of MB-DMD and MB-LMNβ1, were evaluated. For proliferation assessment, we used the CFSE assay, which revealed no significant differences between MB-DMD, cultured on collagen I or on different types of hydrogel (Fig. 2A). In case of MB-LMNβ1 we found that a significantly higher proportion of cells cultured on R1 or R1-SDF1-X or R4-IL4-Y underwent two or more divisions compared to control cells cultured on collagen I, and, at the same time, a lower proportion of cells after one division was found in these samples (Fig. 2B). Furthermore, a higher fraction of cells cultured on the R4-RGD hydrogel underwent two or more divisions compared to control cells (Fig. 2B). The culture on R1, R1-SDF1-X, R4-RGD, or R4-IL4-Y-coated surfaces promoted MB-LMNβ1 proliferation, manifested by the cells undergoing 2 or more divisions, compared to cells cultured on collagen I, and R1-RGD hydrogel. Interestingly, this effect was not observed in the case of MB-DMD (Fig. 2A).

Next, we evaluated the hydrogels’ impact on cell survival by assessing the proportion of apoptotic cells. We found no significant differences between all samples for both analysed cell lines, including control cells cultured on collagen I (Fig. 2C and D). Subsequently, we focused on the hydrogels’ influence on the myogenic differentiation of MB-DMD and MB-LMNβ1. The cells were cultured for 3 days in a SKM02 medium followed by a 6-day culture in myotube induction medium (SKM03). The cells were fixed, stained, and the fusion index was determined, by establishing the proportion of nuclei in myotubes to all nuclei. Myoblasts underwent successful myogenic differentiation manifested by the formation of elongated myotubes when cultured on any type of hydrogel tested as well as on collagen I (Fig. 2E and F). In the case of MB-DMD the effectiveness of myogenic differentiation was significantly higher after culture on R1-SDF1-X or R4-IL4-Y hydrogels, compared to cells cultured on collagen I, as well as R1, R1-RGD, and R4-RGD (for cells cultured on R4-IL4-Y) (Fig. 2G). Thus, modification of hydrogels with SDF1 or IL4 mimicking peptides clearly promoted myogenic differentiation of MB-DMD, but no MB-LMNβ1 cells, in which case no significant differences in the values of the fusion index were found (Fig. 2H).

The effective evaluation of myogenic differentiation, besides showing fusion index values, requires additional confirmation of the findings. Therefore, we determined the expression level of selected myogenic markers (*MYOD*,* MYOG*,* MYHC3*) in cells analysed at day 3 and 9 of the culture. Significantly higher expression of *MYOD* and *MYOG* was found in MB-DMD cultured on R1 hydrogel, compared to control on collagen I on day 3 of the culture (Supplementary Fig. 3A). Additionally, MB-DMD cultured on R4-RGD hydrogel were characterized by elevated levels of *MYOG* and *MYHC3* compared to both control cells, as well as cells cultured on other hydrogels (Supplementary Fig. 3A). An increased level of *MYHC3* was found for MB-LMNβ1 cultured on R4-RGD hydrogel analysed at day 3 (Supplementary Fig. 3B). The expression of *MYOD* and *MYOG* was also significantly higher in these cells, but only when compared to control cells cultured on collagen I (Supplementary Fig. 3B). Furthermore, *MYOD* expression was elevated in MB-LMNβ1 cultured on R4-IL4-Y hydrogel compared to control (Supplementary Fig. 3B). On day 9 no significant changes in myogenic marker expression were found between all analysed samples (Supplementary Fig. 3C, 3D). Together, these results indicate that differences in myogenic marker expression observed in myoblasts cultured on different types of hydrogels were temporal, and all of them supported progression of myoblast differentiation, which resulted in successful myotube formation.

### Migration and non-specific differentiation of hiPSC-derived myoblasts cultured on hydrogels modified with IL4 and SDF1 mimicking peptides

**Fig. 3:**
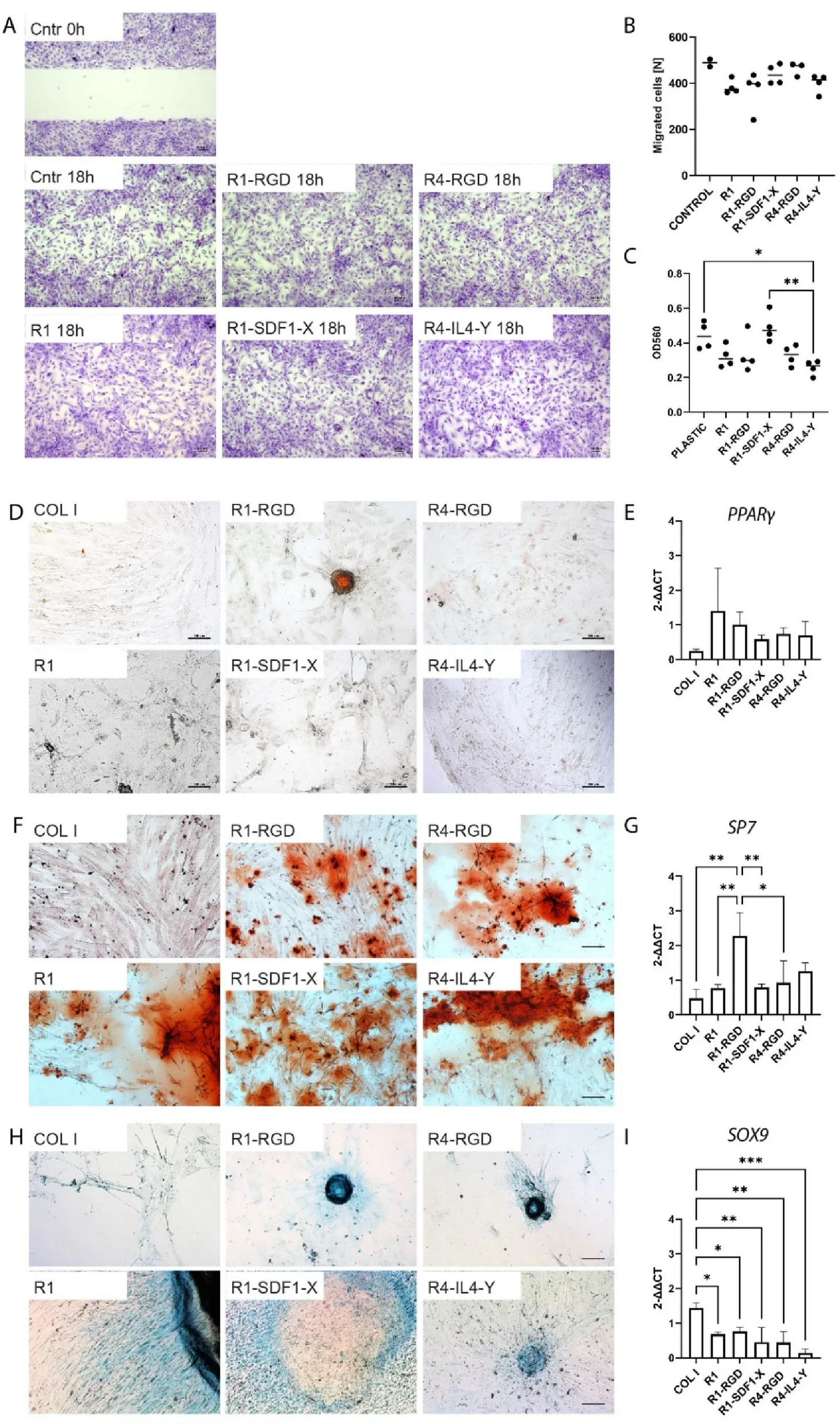
Assessment of migration and undesired differentiation of hiPSC-derived myoblasts cultured on different hydrogels. **A**. Representative images of the scratch wound healing assay after 18 h of MB-DMD culture on hydrogel-coated surfaces or on plastic in the control variant; **B**. Quantification of the scratch wound healing assay. Data are shown as a number of cells that migrated into the wound area in four independent replicates; **C**. Colorimetric migration assay of MB-DMD cultured on collagen I or on peptide functionalised hydrogels after 18 h of culture. The OD was measured at 560 nm. Data are shown for four independent replicates; *p ≤ 0.05, **p ≤ 0.01; **D**. Representative photos of MB-DMD after 14 days of culture in adipogenesis inducing medium on peptide-functionalised hydrogels or on collagen I. Lipid droplets stained with Oil Red O. Scale bar − 100 μm; **E**. qRT-PCR analysis of *PPARγ* mRNA expression in MB-DMD after 14 day culture in adipogenesis inducing medium, on different tested hydrogels or collagen I. **F**. Representative photos of MB-DMD after 7 days of culture in osteogenesis inducing medium on collagen I or hydrogel coated surfaces. Calcium residues visualised with Alizarin Red S. Scale bar − 100 μm; **G**. qRT-PCR analysis of *SP7* mRNA expression in MB-DMD after a 7 day culture in osteogenesis inducing medium, on collagen I or hydrogel-coated surfaces. **H**. Representative photos of MB-DMD after 21 days of culture in chondrogenesis-inducing medium on collagen I or hydrogel coated surfaces. Acidic polysaccharides in cartilage stained with Alcian Blue. Scale bar − 100 μm; **I**. qRT-PCR analysis of *SOX9* mRNA expression in MB-DMD after a 21 day culture in chondrogenesis inducing medium, on hydrogel-coated surfaces. For **E**, **G** and **I** - mean RQ (2^−ΔΔCT^) values with standard deviation from at least 3 experiments are shown. Myoblasts cultured on collagen I served as a reference sample. *p ≤ 0.05, **p ≤ 0.01, ***p ≤ 0.001

Because the ability to migrate is an essential feature of functional myogenic cells, as well as crucial for effective cell therapy designed for skeletal muscles, in the next experiments we investigated this property in MB-DMD cultured on tested hydrogels or on plastic. In this and next experiments we used only one type of myoblasts, i.e., MB-DMD, as these cells were characterized by better myogenic differentiation compared to MB-LMNβ1 (Fig. 2G and H). Scratch assay revealed that cells pre-treated with all analysed hydrogels, as well as collagen I, were able to migrate. The assay was designed to evaluate whether prior culture in the presence of the tested hydrogels affects the migratory potential of MB-DMD cells, however, no significant differences were found (Fig. 3A and B). Therefore, we undertook an alternative approach and used a colorimetric cell migration assay, which revealed that MB-DMD cultured on a R4-IL4-Y hydrogel migrated less efficiently than those cultured on R1-SDF1-X or on plastic, but comparable to all other variants of the experiment (Fig. 3C).

Different studies indicate that myoblasts and SCs, which serve as a source of myoblasts in growing and regenerating muscles, can convert into progenitors responsible for the formation of heterotopic ossification in skeletal muscles and surrounding tissues [49–53]. For this reason, we verified whether hiPSC-derived myoblasts underwent differentiation other than myogenic, i.e., adipogenic, osteogenic or chondrogenic. Oil Red O stained virtually any or only single cells that originated from MB-DMD cultured on indicated hydrogels or collagen I, in a medium promoting adipogenesis (Fig. 3D). Also, the level of *PPARγ*, i.e., an adipogenic marker, was low and uniform across all analysed samples (Fig. 3E). The calcium deposition assessed by Alizarin Red S staining was similar for all variants of the experiments, except for MB-DMD cultured in collagen I, which were characterized by lower level of deposition detected (Fig. 3F). The expression of the osteogenic marker, *SP7*, was consistent across all the samples, except for cells cultured on the R1-RGD hydrogel which were characterised by significantly higher levels of this mRNA (Fig. 3G). Finally, Alcian Blue staining revealed a limited level of acidic polysaccharide accumulation of MB-DMD cultured on R1-RGD, R4-RGD, and R4-IL4-Y, as well as on collagen I (Fig. 3H). Furthermore, the expression of the chondrogenic marker, *SOX9*, was low in all samples analysed, however a significantly higher level of this marker was found in MB-DMD cultured on collagen I than in all other variants (Fig. 3I).

The obtained results indicated that all types of hydrogels tested by us supported the migration of hiPSC-derived myoblasts and did not promote adipogenic differentiation. Although positive Alizarin Red S staining was observed in cells cultured on all hydrogel variants, this was not accompanied by a consistent increase in the expression of the osteogenic marker *SP7*, with a significant increase detected only in cells cultured on the R1-RGD hydrogel. Therefore, the positive Alizarin Red S staining is unlikely to reflect hydrogel-induced osteogenic differentiation and may instead be attributable to residual calcium ions originating from the culture medium or the hydrogel gelation process. However, the expression level of *SOX9* was significantly reduced after exposure to all tested hydrogels, compared to control on collagen I.

### Changes in the transcriptome of human iPSC-derived myoblasts cultured on hydrogels modified with IL4 and SDF1 mimicking peptides

**Fig. 4:**
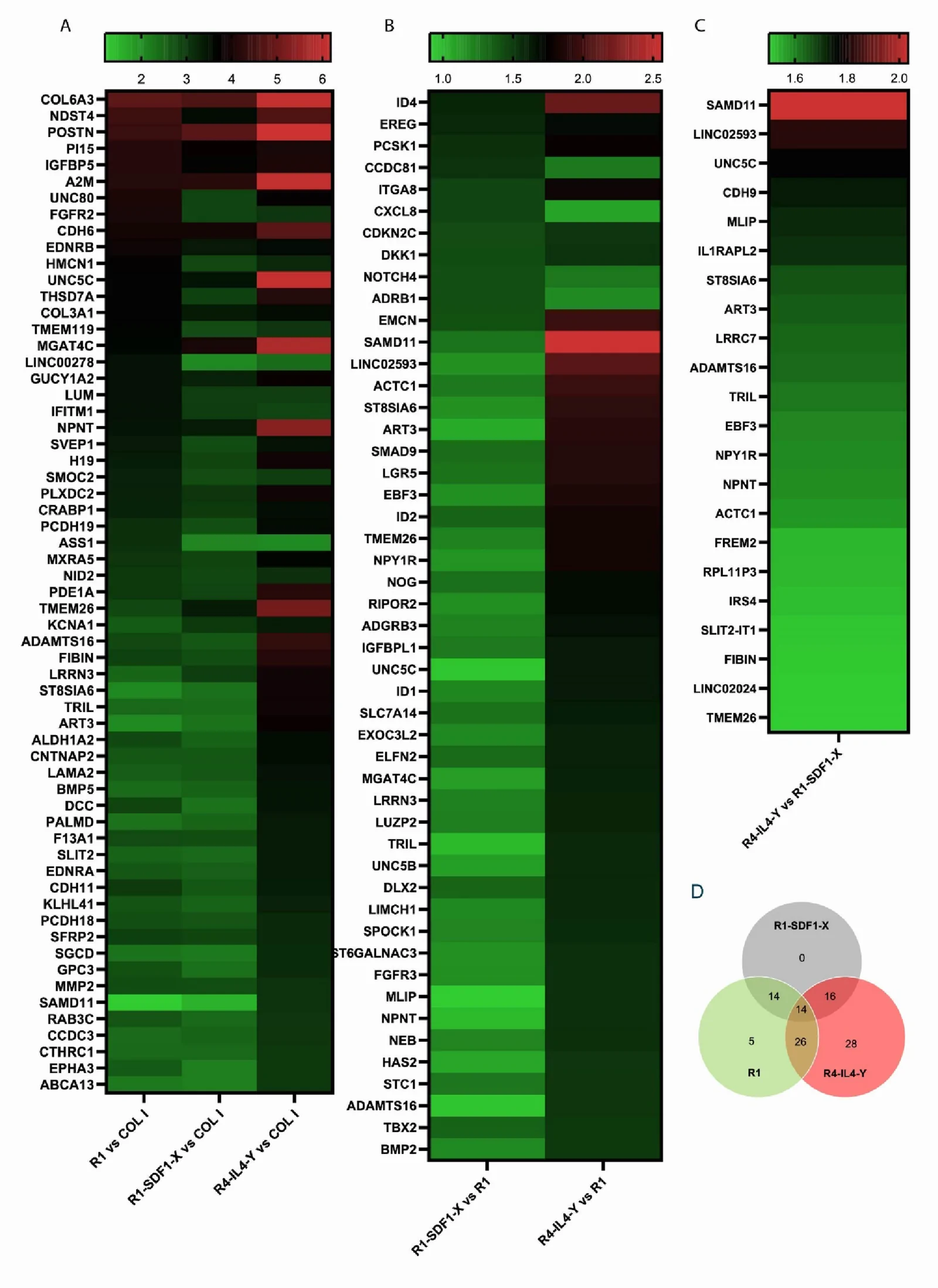
Heat maps and Venn diagram of transcripts up-regulated in hiPSC-derived myoblasts cultured on different types of hydrogels. **A**. Heat map of transcripts up-regulated in myoblasts cultured on indicated hydrogels compared to control cells cultured on collagen I (R1 vs. COL I, R1-SDF1-X vs. COL I, R4-IL4-Y vs. COL I). The list includes transcripts that were characterized by fold-change > 3 at least in one of the indicated comparisons (R1 vs. COL I, R1-SDF1-X vs. COL I, R4-IL4-Y vs. COL I). **B**. Heat map of transcripts up-regulated in myoblasts cultured on indicated hydrogels compared to cells cultured on control hydrogel (R1-SDF1-X vs. R1, R4-IL4-Y vs. R1). The list includes transcripts which were characterised by fold-change > 1.5 at least in one of the indicated comparisons (R1-SDF1-X vs. R1, R4-IL4-Y vs. R1). **C**. Heat map of transcripts up-regulated in myoblasts cultured on R4-IL4-Y compared to cells cultured on R1-SDF1-X hydrogel. The list includes genes which were characterised by fold-change > 1.5. **D**. Venn diagram presents overlapped up-regulated transcripts between myoblasts cultured on different hydrogel types (R1, R1-SDF1-X, R4-IL4-Y)

To better assess the influence of the hydrogels tested on hiPSC-derived myoblasts, we performed a transcriptome analysis by next generation RNA sequencing using MB-DMD cultured on collagen I (control), R1, R1-SDF1-X, or R4-IL4-Y hydrogels. By analysing fold changes, we found numerous differentially expressed transcripts in MB-DMD cultured on tested surfaces (Supplementary Table 2). The annotations of gene ontology (GO) performed using Metascape tool [54] revealed that significantly up-regulated transcripts in analysed cells were associated with processes such as ECM formation (for cells cultured on all types of hydrogels tested), adhesion (cells cultured on R1 or R4-IL4-Y hydrogels) or muscle cell development and differentiation (for cells cultured on all types of tested hydrogels; Supplementary Fig. 4; [54]). As no, or limited, information was found for down-regulated transcripts, we further focused on those which expression was elevated. Thus, we detailly followed the differences in transcript expression found in MB-DMD cultured on hydrogels compared to those cultured on collagen I (R1 vs. collagen I; R1-SDF1-X vs. collagen I, R4-IL4-Y vs. collagen I) as well as between cells cultured on hydrogels modified with SDF1 or IL4 peptides, in comparison to cells cultured on control hydrogel (R1-SDF1-X vs. R1; R4-IL4-Y vs. R1; Fig. 4A and B; Supplementary Table 2). Since our main objective was to precisely describe the changes in MB-DMD induced by each type of tested hydrogel (R1, R1-SDF1-X, or R4-IL4-Y), we focused on the transcripts which expression was specifically altered in cells by each of them (Fig. 4D), compared to cells cultured on collagen I (Supplementary Table 3) or control hydrogel (Supplementary Table 4). We also compared and listed all the transcripts which were up-regulated in cells cultured on R4-IL4-Y hydrogel, compared to more than one other sample (versus collagen I, R1, and/or R1-SDF1-X; Supplementary Table 5). Next, we carefully checked the characteristics of all listed transcripts, using *inter alia* ShinyGO 0.82 and STRING [55–57]. Based on that, we have indicated the list of 17 transcripts that were elevated in cells cultured on different types of hydrogels, which are known to play an important role and/or are involved in pathways crucial for myogenic cell development, maintenance, and differentiation (Supplementary Table 6). The listed transcripts were subjected to STRING analysis with results shown in Supplementary Tables 7 and Supplementary Fig. 5.

**Fig. 5:**
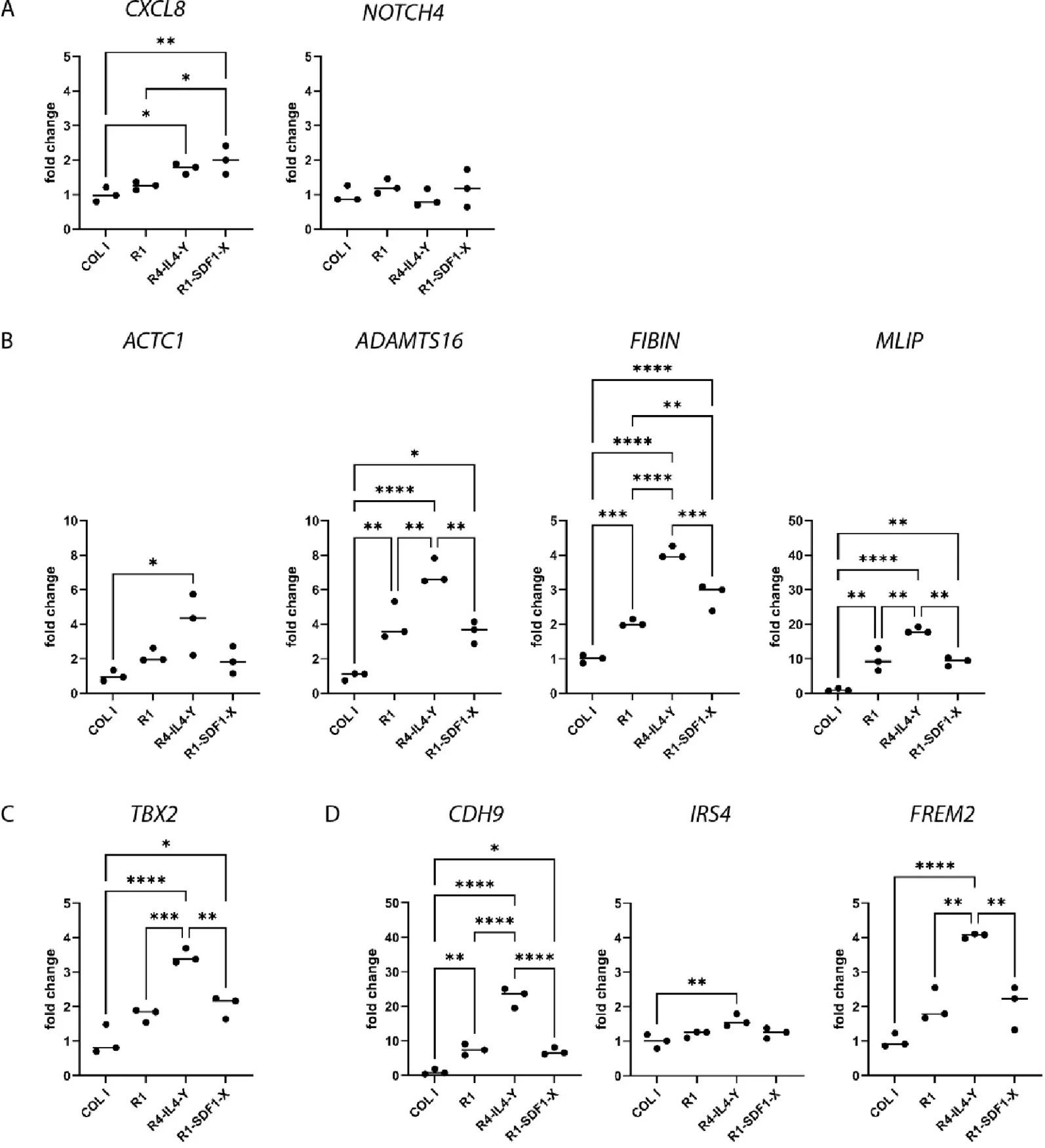
qRT-PCR analysis of transcripts selected on the basis of NGS analysis. mRNA expression was determined in MB-DMD after 3 days of culture on collagen I (COL I, control) or hydrogel-coated surfaces. Data are shown as fold change compared to cells cultured in collagen I. * *p* ≤ 0.05, ** *p* ≤ 0.01, *** *p* ≤ 0.001, **** *p* ≤ 0.0001. **A**. Transcripts found by NGS analysis to be elevated in cells cultured on R1-SDF1-X to R1. **B**. Transcripts found by NGS analysis to be elevated in cells cultured on R4-IL4-Y to other variants. **C**. Transcripts found by NGS analysis to be elevated in cells cultured on R4-IL4-Y to R1. **D**. Transcripts found by NGS analysis to be elevated in cells cultured on R4-IL4-Y to R1-SDF1-X

Next, the expression level of the transcripts revealed by NGS analysis as differentially expressed in MB-DMD cultured on different types of hydrogels, was evaluated by RT-qPCR (Fig. 5). As indicated by the NGS results, a significantly higher level of *CXCL8* was found in myoblasts cultured on the R1-SDF1-X hydrogel (Fig. 5A), however RT-qPCR analysis revealed that it was also enhanced in MB-DMD cultured on R4-IL4-Y hydrogels compared to control cells (Fig. 5A). The second transcript selected on the NGS results, *NOTCH4*, was expressed at a similar level in all types of samples (Fig. 5A). Next, we found that mRNAs encoding *ACTC1*, *ADAMTS16*, *FIBIN*, and *MLIP*, i.e., genes that were indicated by NGS analysis as significantly up-regulated in cells cultured on the R4-IL4-Y hydrogel compared to other experiment variants (collagen I, R1, R1-SDF1-X), were indeed significantly elevated in such cells (Fig. 5B). The exception was found for *ACTC1*, in which case the significantly higher expression was also detected in cells cultured on R1 or R1-SDF1-X hydrogels, compared to control cells propagated on collagen I (Fig. 5B). The *TBX2* mRNA was significantly elevated in cells cultured on R4-IL4-Y, compared to other samples, as well as in cells propagated on R1-SDF1-X hydrogel, in comparison to control cells (Fig. 5C). In the case of other transcripts, indicated as up-regulated in cells cultured on the R4-IL4-Y hydrogel compared to R1 (i.e., *BMP2*, *ID1*, *ID2*, *LGR5*, *NEB*, *NOG*, *SMAD9*), we did not notice significant differences in their expression between samples (Supplementary Fig. 6A). However, cells cultured on the R4-IL4-Y hydrogel were characterised by significantly higher levels of *CDH9*, *IRS4* and *FREM2* as indicated by NGS analysis (Fig. 5D). Lastly, we performed Luminex analysis to verify the level of selected proteins, i.e. SDF1, IL4, IL8, VEGF-A, and BMP2, in medium collected from myoblast cultures on tested hydrogels or collagen I. We also evaluated the concentration of these proteins in fresh medium in which hiPSC-derived myoblasts were cultured, i.e., SKM02. The results of the Luminex analysis did not indicate significant differences in either SDF1 or IL8 levels between cells cultured on different surfaces (Supplementary Fig. 6B). The concentration of VEGF-A was lower in the medium collected from the myoblast cultured on R4-IL4-Y or R1-SDF1-X hydrogels compared to media coming from control cells on collagen I, however the difference was not statistically significant (Supplementary Fig. 6B). The level of IL4 and BMP2 was found out of range (too low) in performed analysis (data not shown).

Taken together, hydrogel presence induced changes in the expression level of factors, such as *CXCL8*, *FREM2*, *ADAMTS16*, *CDH9*, *IRS4*, *FIBIN*, or *MLIP* involved in angiogenesis, inflammatory mediation, cell adhesion, neurogenesis, and regulation of myogenic program in myoblasts derived from human iPSCs, which could be beneficial considering myogenic/regenerative potential of these cells.

## Discussion

hiPSCs serve as a promising source of myogenic cells that could be used to support the reconstruction of injured or dystrophic skeletal muscle. Various methods have already been explored to induce myogenic differentiation in hiPSCs including the three-step protocol used in the present study [22]. Here, RADA16-I hydrogels modified with custom-designed IL4 or SDF1 mimicking peptides were tested to establish an optimal environment that would support the myogenic/regenerative potential of hiPSC-derived myoblasts. The hydrogels were applied as thin bioactive coatings for *in vitro* experiment. The hydrogels were designed using RADA16-I scaffold, i.e., an example of SAP-based hydrogels, which are emerging as promising biomaterials both for cell propagation and transplantation, due to their ability to mimic the extracellular matrix [58]. SAP-hydrogels were shown to promote a critical step in muscle repair, i.e., the alignment and differentiation of myoblasts into mature myotubes *in vitro* [59, 60]. For instance, SAP-nanofiber hydrogel based on self-assembling backbone Ac-(RADA)_4_-NH_2_ (RAD) dual-functionalized with laminin-derived motif IKVAV (IKV) and a BDNF-mimetic peptide enhanced myoblast proliferation and differentiation [61]. Detailed analyses performed by us showed that the impact of hydrogels tested on myoblast proliferation or fusion differed depending on the cell line used. However, it should be mentioned that variability between hiPSC lines may arise from differences in donor genetic background, reprogramming-associated alterations, epigenetic status, and intrinsic differentiation abilities [62]. Therefore, an increased proliferation of MB-LMNβ1, but not MB-DMD, was observed as a response to culture on hydrogels. This phenomenon was independent of the presence of either IL4 or SDF1 mimicking peptides. Thus, we suggested that hydrogel without functionalization could be the supportive substrate for MB-LMNβ1 culture. Moreover, in case of cell culture on collagen I, it was revealed that proliferation of MB-LMNβ1 was decreased compared to MB-DMD. Thus, the use of hydrogels could be beneficial for cell lines with characteristics similar to MB-LMNβ1. This finding was in some way in agreement with the results of our previous study showing that the presence of IL4 or SDF1 mimicking peptides, without hydrogels, did not impact the proliferation of many cell types such as human fibroblasts, myoblasts, and bone marrow stromal cells [34]. Importantly, hydrogels did not induce cell apoptosis, which allows them to be considered suitable for transplantation applications.

The efficiency of myogenic cell differentiation is one of the most important factors that indicate cells’ potential to support skeletal muscle regeneration. Hydrogels modified with IL4 or SDF1 mimicking peptides impaired neither MB-LMNβ1 nor MB-DMD fusion, as tested cells were able to form myotubes. However, the effectiveness of MB-DMD myotube formation was higher in the presence of hydrogels modified with IL4 or SDF1 mimicking peptides, compared to control. Such an effect was not observed in the case of unmodified hydrogels, thus, we concluded that the presence of IL4 or SDF1 mimicking peptides enhanced MB-DMD fusion. We further suggested that the functionalized hydrogels could support MB-DMD differentiation. Importantly, GO analysis showed that transcripts involved in muscle cell differentiation were elevated in myoblasts cultured in the presence of hydrogels modified with either IL4 or SDF1 mimicking peptides, compared to unmodified hydrogels. This is in line with the results indicating that IL4 is an important factor responsible for the recruitment of myoblasts to form myotubes *in vitro* [63]. Furthermore, the reduced area of myofibers was detected in skeletal muscles of IL4-/- mice, confirming the crucial role of IL4 signalling in myogenic cell fusion with myofibers* in vivo*. Using mouse C2C12 cells, it was also shown that the IL4/IL4Rα axis enhanced cell fusion and differentiation in a MYOD, myogenin, and myomerger dependent manner [4]. In turn, the impact of SDF1 on myoblast fusion is unclear. Some lines of evidence suggest that SDF1 inhibited the differentiation of both C2C12 and primary murine myoblasts, decreasing MYOD, MYHC, and myogenin levels [64]. However, it was also shown that SDF1 treatment led to MYHC accumulation and induced C2C12 myoblasts and primary murine myoblasts to form myotubes [65]. Our previous studies showed that SDF1 treatment could improve fusion of bone marrow stromal cells with myotubes [66], suggesting that the impact of SDF1 on cell fusion could depend on cell types.

Another factor determining the regenerative potential of cells is their ability to migrate. SDF1 is a well-known chemoattractant and many research groups evaluated this aspect of SDF1 activity [9, 11, 12, 67–69]. In our previous study we noticed that SDF1, as well as IL4 mimicking peptides, were able to induce migration of human fibroblasts, and myoblasts [34]. SDF1 mimicking peptide also improved the migration of bone marrow stromal cells. In the current study, using a wound healing assay, we showed that the migration of MB-DMD cultured on hydrogels modified with IL4 or SDF1 peptides was not changed. However, in the transwell colorimetric cell migration assay, MB-DMD migrated significantly more efficiently in culture on R1-SDF1-X and on plastic, compared to those cultured on R4-IL4-Y. The significant inhibition of migration towards hydrogel modified with IL4 peptide was confirmed by GO analysis, revealing that transcripts elevated in such cells were connected *inter alia* with negative regulation of migration. Interestingly, IL4 was previously shown to favour human myogenic cell migration and its interaction with newly formed myotubes [3]. However, it was also shown that IL4 did not influence the migration of primary prostate cancer cells [70] or vascular smooth muscle cells [71] and could also inhibit migration of other cells, e.g., human keratinocytes [72].

Considering the possibility of using hydrogels *in vivo*, it is important to determine if the presence of such scaffolds impacts undesired cell differentiation. Importantly, hydrogels modified with IL4 or SDF1 mimicking peptides did not enhance MB-DMD neither osteogenesis nor adipogenesis. However, in the presence of hydrogels, the up-regulation of *OSF-2* (osteoblast-specific factor) and *IGFBP5* (insulin-like growth factor binding protein 5) was revealed by NGS analysis. *OSF-2* is a transcription factor which regulates osteoblast differentiation [73] and *IGFBP5* is known as a pro-osteogenic factor [74]. Importantly, hydrogels inhibited the expression of chondrogenic marker *SOX9* in MB-DMD, which could be related to *NOGGIN* upregulation found in NGS analysis. *NOGGIN* inhibits chondrocyte and osteocyte differentiation through negative regulation of TGF-β/BMP signalling [75]. Thus, hydrogels tested by us do not pose the risk of inducing unwanted differentiation processes; however, *in vivo* studies are needed to exclude such risk.

In addition to the above-mentioned results, NGS analysis revealed a significant number of transcripts which were up-regulated or, to lesser extent – down-regulated in MB-DMD, in response to culture on different types of hydrogels. Some changes in transcript expression were common for cells cultured on all tested hydrogels (R1, R1-SDF1-X, R4-IL4-Y), indicating that they were not specifically induced by a particular hydrogel type, or hydrogel modification. Among them were *POSTN*, encoding periostin, which serves as a ligand for integrins and supports cell adhesion and migration [76], *NPNT* which product plays a role in cell adhesion, spreading and survival through the binding of several integrins, or *IGFBP5*, which encodes multifunctional protein that influences among many proliferation, adhesion, differentiation, and apoptosis in a cell-type specific manner [77]. The range and composition of transcripts which expression was significantly changed and altered among MB-DMD cultured on particular hydrogel types, indicated that each hydrogel, or peptide released from them, induced specific cell response. Although GO analysis revealed that all types of tested hydrogels influenced expression of transcripts connected to the same, or similar biological processes, such as formation of extracellular matrix, or cell morphogenesis, they also had a specific, distinguishable impact. For example, the changes in the transcriptome induced by control R1 hydrogel were connected *inter alia* with potential osteogenesis regulation, while R4-IL4-Y elevated expression of several transcripts that are typically associated with neurogenic or epithelial pathways. This is an important issue in case of derivatives obtained from pluripotent iPSCs which initially have a potential to differentiate into cell types originating from all germ layers. Thus, it cannot be definitely excluded that in response to strong inducers, iPSC derivatives could still change their fate.

Detailed analysis of transcriptome differences showed that among transcripts which expression was specifically changed in the response to R1-SDF1-X hydrogel, compared to R1 hydrogel, were *CXCL8* and *NOTCH4*. Both transcripts encode factors essential for proper skeletal muscle function. CXCL8 acts as a chemotactic factor that mediates inflammatory response in injured muscles, serves as a potential angiogenic factor, and is involved in controlling cell fate [78]. It is released by contracting skeletal muscles and acts locally within the muscle microenvironment, where it may support tissue remodelling and repair processes [79]. Importantly, CXCL8 promotes myotube growth, by increasing myotube length and diameter, activating AKT signalling pathways, reducing the expression of proteolysis-associated markers, e.g., atrogin and MuRF1, as well as supporting myogenic differentiation [80]. In turn *NOTCH4* is engaged in cell-fate determination. Next, among transcripts specifically up-regulated by the R4-IL4-Y hydrogel were *inter alia* those connected with skeletal muscle development and maintenance such as *LGR5*, which product is involved in the canonical WNT signalling pathway, already mentioned *NOGGIN*, encoding protein expressed during muscle development and acting as a BMP inhibitor, or *NEBULIN* which product localises to thin filament in skeletal muscles and regulates actin-myosin interactions [81–83]. GO analysis stayed in accordance with the observed differences in the elevated transcript sets in MB-DMD, in response to functionalised hydrogels showing that genes upregulated in R1-SDF1-X variant were related to, e.g., muscle cell differentiation and angiogenesis, while in R4-IL4-Y variant to, e.g., muscle structure development and negative muscle differentiation. The results of the NGS analysis allowed us for setting the list of common transcripts that were elevated in MB-DMD cultured on the R4-IL4-Y hydrogel compared to any other variant (cells cultured on collagen I, R1 and/or R1-SDF1-X hydrogel). Among them was the muscular LMNA interacting protein, i.e. *MLIP*, required for myoblast differentiation in myotubes and acting as a potential transcriptional regulator of the myogenic program, or *ACTC1*, i.e., cardiac muscle alpha actin, serving as a major constituent of the contractile apparatus in muscle tissues [84, 85].

RT-qPCR analysis, which followed NGS, confirmed that MB-DMD cultured on R1-SDF1-X hydrogel were indeed characterised by significantly higher level of *CXCL8* expression, compared to those cultured on the R1 hydrogel as well as control ones, propagated on collagen I. An elevated level of *CXCL8* was also found in MB-DMD cultured on R4-IL4-Y hydrogel, compared to control ones. Stimulation of *CXCL8* expression by IL4 has already been described *inter alia* for endothelial cells [86], while the linkage between *CXCL8* and SDF1 activity was shown only for pancreatic cancer cells, in which SDF1 release caused fibroblasts to produce *CXCL8* and attract cancer cells [87]. Under experimental conditions used by us, the release of SDF1 peptide from the hydrogel could stimulate *CXCL8* expression in hiPSC-derived myoblasts. This feature could be beneficial in the case of transplantation of such cells pre-treated with this hydrogel, as CXCL8 is a known mediator of the inflammatory response in injured muscles, as a well as a potential angiogenic factor, that could be helpful during muscle reconstruction [88]. It was also shown as a positive regulator of skeletal muscle cell differentiation, therefore its expression in hiPSC-derived myoblasts may reflect, and accompany, their myogenic identity. An inverted picture was obtained for *TBX2*, which encodes the T-box transcription factor, and which expression was significantly elevated in MB-DMD cultured on R4-IL4-Y hydrogel, compared to all other samples. It was also higher in cells propagated on R1-SDF1-X hydrogel, compared to control cells. TBX2 is associated with numerous signalling pathways such as BMP, TGFβ, WNT, and FGF, and thus is engaged in regulation of cell proliferation and tissue patterning during organogenesis. TBX2 was also shown to control the cell cycle in skeletal muscle through interaction with MYOD and cell cycle regulators, p21 and p14 [89]. However, the exact mechanism of its action, as well as its possible connection with SDF1 or IL4, have not been described yet.

Human iPSC-derived myoblasts cultured on hydrogel modified with IL4 mimicking peptide were also characterised by significantly elevated levels of *FREM2* compared to all other samples as well as *IRS4* and *ACTC1* in comparison to control samples. FREM2, i.e. FRAS1-related extracellular matrix protein 2, is described as a factor that forms a complex with other ECM proteins, FREM1 and FRAS1, and plays an important role in cell adhesion and cell membrane integrity [90, 91]. In turn, *ACTC1* and *IRS4* were also significantly increased in myoblasts cultured in the presence of R4-IL4-Y hydrogel, but only compared to control cells. *ACTC1* encodes the predominant striated α-actin isoform in the heart, but it is also expressed in developing and early adult skeletal muscles [85]. It represents a therapeutic target for disease caused by a mutation in *ACTA1* encoding skeletal muscle α-actin which generally leads to a severe skeletal muscle phenotype with reduced movement at birth and death within the first year of life [92]. Transgenic overexpression of *ACTC1* in postnatal skeletal muscle could effectively compensate for absent or mutant *ACTA1* [93–95]. Its elimination in bovine myoblasts resulted in the reduction in myotubes’ size and number, as well as the decrease in myogenic marker expression [96]. The potential connection of ACTC1 with IL4 remains unknown, however, identification of the factors directly or indirectly controlling its expression, would be important for possible treatments of cardiac or skeletal muscle diseases in the future. Next, IRS4 is known to be involved in tyrosine kinase signalling, also in response to IL4 activity [97]. It acts as a regulator of BMP signal transduction, leading to its repression in mouse C2C12 myoblasts and activation of PI3K/AKT pathway [98]. For this reason, it is believed to serve as a molecular switch between BMP and AKT signalling, and to be particularly critical in the myoblasts, controlling their differentiation and final commitment. Alterations in the BMP pathway, i.e., up-regulation of *BMP2* and *SMAD9* expression, as well as their targets – *ID1* and *ID2*, were indicated by NGS analysis in myoblasts cultured on R4-IL4-Y, compared to those propagated on R1. However, neither RT-qPCR nor Luminex analysis confirmed such an effect. This could result from the above-mentioned increase in *IRS4* expression in response to IL4, which could lead to inhibiting BMP signalling and progression of myogenic differentiation, which occurred successfully in hiPSC-derived myoblasts, analysed by us.

NGS analysis also indicated that myoblast propagation on R4-IL4-Y hydrogel resulted in up-regulation of *CDH9*, compared to cells cultured on R1-SDF1-X, as well as *ADAMTS16*,* FIBIN* and *MLIP*, in comparison to cells cultured on all other surfaces (collagen I, R1, R1-SDF1-X hydrogel), what was confirmed by RT-qPCR analysis. *CDH9*,* ADAMTS16* and *MLIP* were also expressed at a higher level in cells cultured on other hydrogels, i.e., R1 and R1-SDF1-X, but only compared to control MB-DMD propagated on collagen I, and at significantly lower level than in cells cultured in the presence of hydrogel modified with IL4 mimicking peptide. *FIBIN* expression was also up-regulated in MB-DMD cultured in the presence of R1 or R1-SDF1-X hydrogel, but in this case the expression level in cells cultured on the R1-SDF1-X hydrogel was higher not only in comparison to control cells, but also those propagated on the R1 hydrogel. CDH9 is known to regulate synapse formation [99], but it was also detected in developing and mature skeletal muscle [100, 101]. It was indicated to bind to adiponectin, however, little is known about its actual role in the functioning of skeletal muscle or myogenic cells. Even less information is available on *ADAMTS16*, which is mostly known to be involved in renal and gonadal development, as well as blood pressure regulation [102]. Experiments performed with renal tubule-derived, epithelial MDCKI cells, showed that ADAMTS16 cleaves fibronectin, leading to the inhibition of its maturation and up-regulation of *MMP3* and *MMP14* expression. We have indeed noticed this effect in MB-DMD cultured on R4-IL4-Y, in the case of *MMP14* but not *MMP3*. In contrast to the factors mentioned above, MLIP has been described as a factor ubiquitously and abundantly expressed in skeletal muscles. It acts as a transcription factor that regulates the myogenic program through its association with chromatin and is required for myoblast differentiation into myotubes [103]. Blocking *MLIP* expression in C2C12 myoblasts negatively impacted MRFs, inhibited myoblast differentiation and myotube formation. MLIP is also engaged in organising the position of myonuclei which number in myotubes and muscle fibers is increased by well described action of IL4 [63]. The last transcript significantly increased in myoblasts cultured on R4-IL4-Y hydrogel, *FIBIN*, encodes the factor which was initially described as expressed in the mesoderm and necessary for fin formation [104]. Later, using a knock-out mouse model, it was also shown that FIBIN is crucial for proper muscle fiber development during embryogenesis and control of skeletal muscle mass in adulthood. The analysis of mouse C2C12 myoblasts revealed that *FIBIN* inhibits their proliferation and is crucial for their differentiation, as cells lacking this gene had almost no ability to form myotubes. Recently, FIBIN was also shown to regulate mitochondrial function in myoblasts by binding to mitochondria related proteins such as RRBP1, ATP5F1B, and TPI1 [105]. Altogether, hydrogels tested by us induced changes in the transcriptome of hiPSC-derived myoblasts involving factors engaged in angiogenesis and inflammatory mediation (*CXCL8*), cell adhesion (*FREM2*) and increase in MMP expression (*ADAMTS16*), as well as neurogenesis (*CDH9*), and progression and maintenance of myogenic differentiation (*IRS4*,* FIBIN*,* MLIP*).

## Conclusions

The hydrogels tested by us turned out to secure a supportive environment for myoblasts obtained from human iPSCs, as they enabled proper proliferation, migration, and myogenic differentiation of these cells. In the case of hydrogels functionalised with IL4 or SDF1- derived peptides their beneficial impact is underlined by an increased fusion index. Moreover, none of the tested hydrogels induced neither apoptosis nor non-specific differentiation of the hiPSC-derived myoblasts. Hydrogel-induced changes in the transcriptome of hiPSC-derived myoblasts are beneficial for their regenerative potential, as they concerned factors regulating *inter alia* angiogenesis, inflammatory mediation, neurogenesis, and myogenic differentiation. Hopefully, our ongoing *in vivo* studies confirm that the above-mentioned effects translate to significant benefits observed during the reconstruction of muscles transplanted with hydrogels functionalised with either IL4 or SDF1- derived peptides, and hiPSC-derived myoblasts.

## Supplementary Information

Below is the link to the electronic supplementary material.

Supplementary Material 1.

## Data Availability

The data supporting the findings of this study are available within the article and its supplementary materials. The raw sequence data have been archived in University of Warsaw Repository [https://danebadawcze.uw.edu.pl/dataset.xhtml? persistentId=doi:10.58132/XYKGK1](https:/danebadawcze.uw.edu.pl/dataset.xhtml? persistentId=doi:10.58132/XYKGK1) .

## Abbreviations

CFSE: Carboxyfluorescein succinimidyl ester
DIC: Diisopropylcarbodiimide
ESCs: Embryonic stem cells
IL4: Interleukin 4
iPSCs: Induced pluripotent stem cells
hiPSC: Human induced pluripotent stem cells
hiPSC-MBs: hiPSC-derived myoblasts
HPLC: High-performance liquid chromatography
MB-DMD: Myoblasts derived from hiPSC-DMD line
MB-LMNβ1: Myoblasts derived from hiPSC-LMNβ1 line
MMPs: Metalloproteinases
MYHC: Myosin heavy chains
qPCR: Quantitative polymerase chain reaction
PFA: Paraformaldehyde
R1: Unfunctionalised RADA16-I hydrogel
SAPs: Self-assembling peptides
SCs: Satellite cells
SDF1: Stromal derived factor 1
SPPS: Solid phase peptide synthesis
TFA: Trifloroacetic acid
TIS: Triisopropylsilane
VML: Volumetric muscle loss
