# Supplementary material for: Hydrogels modified with IL4 or SDF1 mimicking peptides serve as a supportive environment for human iPSC-derived myoblasts
Source: Stem Cell Res Ther. 2026 Jul 26;17:377. doi: 10.1186/s13287-026-05192-1 (PMC13689189; doi:10.1186/s13287-026-05192-1)
Supplement: Supplementary file 1 [file 13287_2026_5192_MOESM1_ESM.docx]

**Supplementary Fig. 1.** **Myogenic differentiation of hiPSC-DMD and hiPSC-LMNβ1.**

A. Representative microscope images of the subsequent stages of hiPSC myogenic differentiation analysed: undifferentiated hiPSCs, myoprecursors, myoblasts, and myotubes. Scale bar − 100 µm; B. The expression of *PAX7*, *MYOD*, *MYHC2*, *MYHC3* and *MYHC7* mRNA measured by RT-qPCR in undifferentiated hiPSC-DMD and hiPSC-LMNβ1; mean ΔCT values with standard deviation of at least 3 experiments are shown; stars indicate significant statistical differences between cell lines: ** p ≤ 0.01, **** p ≤ 0.0001, NS - no significant differences. C. Expression of selected myogenic markers in cells at the subsequent stages of iPSC myogenic differentiation: undifferentiated hiPSCs, myoprecursors, myoblasts, and myotubes. The mean values of RQ (2^−ΔΔCT^) values with the standard deviation from at least 3 experiments are shown; undifferentiated iPSCs served as a reference sample. * p ≤ 0.05, ** p ≤ 0.01, *** p ≤ 0.001, **** p ≤ 0.0001.

**Supplementary Fig. 2.** **Expression profile of selected *MMPs* in hiPSC-derived myoblasts.** A. Expression of *MMP2*, *MMP3*, *MMP7*, *MMP9*, *MMP13* and *MMP14* mRNA measured by RT-qPCR in hiPSCS-derived myoblasts (MB-DMD and MB-LMNβ1) cultured on collagen I; mean ΔCT values with standard deviation of at least 3 experiments are shown; ** p ≤ 0.01, NS - no significant differences. B. Expression of MMPs in MB-DMD and MB-LMNβ1 cultured on different hydrogels (R1, R1-RGD, R1-SDF1-X, R4-RGD, R4-IL4-Y) or collagen I (COL I). The mean RQ (2^−ΔΔCT^) values with standard deviation from at least 3 experiments are shown. The myoblasts on day 0 served as a reference sample. Only significant differences between cells cultured on collagen I and hydrogels are shown. * p ≤ 0.05, ** p ≤ 0.01, *** p ≤ 0.001, **** p ≤ 0.0001. C. Summary of changes in MMP expression between myoblasts cultured on different hydrogels in comparison to control myoblasts cultured on collagen I.

**Supplementary Fig. 3.** **Expression of selected myogenic markers in hiPSC-derived myoblasts cultured on different hydrogels.** A. Expression of *MYOD*, *MYOG*, *MYHC3* in MB-DMD cultured for 3 days in SKM02 on different hydrogels (R1, R1-RGD, R1-SDF1-X, R4-RGD, R4-IL4-Y) or in the control variant, on collagen (COL I). B. Expression of myogenic markers in MB-LMNβ1 cultured for 3 days in SKM02 on different hydrogels or on collagen (COL I). C. Expression of myogenic markers in MB-DMD cultured for 3 days in SKM02, followed by 6 days in SKM03 on different hydrogels or collagen (COL I). D. Expression of myogenic markers in MB-LMNβ1 cultured for 3 days in SKM02, followed by 6 days in SKM03 on different hydrogels or collagen (COL I). For A-D: The mean RQ (2^−ΔΔCT^) values with standard deviation of at least 3 experiments are shown. Myoblasts cultured on collagen I served as a reference sample. *p ≤ 0.05, ** p ≤ 0.01, *** p ≤ 0.001.

**Supplementary Fig. 4. Gene ontology (GO) analysis of terms related to certain biological processes for transcripts most significantly up-regulated in cells cultured on different types of hydrogels.** Analysis was performed with Metascape tool (54).

**Supplementary Fig. 5.** **STRING analysis of selected differentially expressed transcripts in hiPS-derived myoblasts cultured on different hydrogel types.** The figure was retrieved from the STRING database together with data shown in Supplementary Table 7 for top 17 transcripts changed as a result of culture of MB-DMD on different hydrogels, listed in Supplementary Table 6.

**Supplementary Fig. 6.** **qRT-PCR and Luminex analysis of selected factors chosen on the basis of NGS analysis.** A. RT-qPCR analysis of *BMP2, ID1, ID2, LGR5, NEB, NOG, SMAD9* mRNA expression was determined in MB-DMD after 3 days of culture on collagen I (COL I, control) or hydrogel-coated surfaces. Data are shown as fold change compared to cells cultured in collagen I. B. Luminex analysis of indicated proteins. The mean value with standard deviation of 4 experiments is shown.

**Supplementary Fig. 7.** A. Representative photos of myotubes formed during MB-DMD culture on indicated surfaces, stained with Giemsa-May Grünwald. Scale bar − 100 µm; B. Representative photos of myotubes formed during MB-LMNβ1 culture on the indicated surfaces, stained with Giemsa-May Grünwald. Higher-magnification images from Figs. 2E and 2F respectively. Scale bar − 100 µm.

**Supplementary Table 1. TaqMan probes used in RT-qPCR analysis.**

| **Gene** | **Name** | **Reference** |
| --- | --- | --- |
| *ACTC1* | Actin, alpha, cardiac muscle 1 | Hs01109515_m1 |
| *ADAMTS1* | ADAM metallopeptidase with thrombospondin type 1 motif 1 | Hs00199608_m1 |
| *BMP2* | Bone morphogenetic protein 2 | Hs00154192_m1 |
| *CDH9* | Cadherin 9 | Hs00940349_m1 |
| *CXCL8* | Chemokine ligand 8 of the C-X-C motif chemokine ligand 8 | Hs00174103_m1 |
| *FIBIN* | Fin bud initiation factor homolog | Hs00831591_s1 |
| *FREM2* | FRAS1-related extracellular matrix protein 2 | Hs01388268_m1 |
| *GAPDH* | Glyceraldehyde-3-phosphate dehydrogenase | Hs04420697_g1 |
| *ID1* | Inhibitor of DNA binding 1, HLH protein | Hs03676575_s1 |
| *ID2* | Inhibitor of DNA binding 2, HLH protein | Hs04187239_m1 |
| *IRS4* | Insulin receptor substrate 4 | Hs00269220_s1 |
| *LGR5* | Leucine-rich repeat containing G protein-coupled receptor 5 | Hs00969422_m1 |
| *MLIP* | Muscular LMNA-interacting protein | Hs00370866_m1 |
| *MMP2* | Matrix metallopeptidase 2 | Hs01548728_m1 |
| *MMP3* | Matrix metallopeptidase 3 | Hs06633340_s1 |
| *MMP7* | Matrix metallopeptidase 7 | Hs01042796_m1 |
| *MMP9* | Matrix metallopeptidase 9 | Hs00957562_m1 |
| *MMP13* | Matrix metallopeptidase 13 | Hs00942584_m1 |
| *MMP14* | Matrix metallopeptidase 14 | Hs01037003_g1 |
| *MYHC2* | Myosin heavy chain 2 | Hs00430042_m1 |
| *MYHC3* | Myosin, heavy chain 3, skeletal muscle, embryonic | Hs01074230_m1 |
| *MYHC7* | Myosin, heavy chain 7, cardiac muscle, beta | Hs01110632_m1 |
| *MYOD* | Myogenic differentiation 1 | Hs00159528_m1 |
| *MYOG* | Myogenin | Hs01072232_m1 |
| *NEB* | Nebulin | Hs00189880_m1 |
| *NOG* | Noggin | Hs00271352_s1 |
| *NOTCH4* | Notch 4 | Hs00965889_m1 |
| *PAX7* | Paired box 7 | Hs01553856_m1 |
| *PPARγ* | Peroxisome proliferator activated receptor gamma | Hs01115512_m1 |
| *SMAD9* | SMAD9 intronic transcript 1 | Hs05006034_g1 |
| *SOX9* | SRY-box 9 | Hs00165814_m1 |
| *SP7* | Sp7 transcription factor | Hs01866874_s1 |
| *TBX2* | T-box 2 | Hs00911929_m1 |

**Supplementary Table 2. Top changes in transcripts revealed by NGS analysis.**

| **Comparison** | **R1  vs COL I** | **R1-SDF1-X vs COL I** | **R4-IL4-Y  vs COL I** | **R1-SDF1-X vs R1** | **R4-IL4-Y  vs R1** | **R4-IL4-Y  vs R1-SDF1-X** |
| --- | --- | --- | --- | --- | --- | --- |
| Differentially expressed transcripts | **37** | **19** | **66** | **42** | **56** | **24** |
| Up-regulated | **31**  COL6A3  NDST4  POSTN  PI15  IGFBP5  A2M  UNC80  FGFR2  CDH6  EDNRB  HMCN1  UNC5C  THSD7A  COL3A1  TMEM119  MGAT4C  LINC00278  GUCY1A2  LUM  IFITM1  NPNT  SVEP1  H19  SMOC2  PLXDC2  CRABP1  PCDH19  ASS1  MXRA5  NID2  PDE1A | **16**  POSTN  COL6A3  A2M  MGAT4C  CDH6  PI15  IGFBP5  NDST4  UNC5C  EDNRB  COL3A1  TMEM26  NPNT  GUCY1A2  PLXDC2  KCNA1 | **56**  POSTN  UNC5C  COL6A3  A2M  MGAT4C  NPNT  TMEM26  CDH6  NDST4  ADAMTS16  PDE1A  FIBIN  THSD7A  IGFBP5  PI15  LRRN3  PLXDC2  H19  ST8SIA6  TRIL  GUCY1A2  ART3  UNC80  MXRA5  EDNRB  PCDH19  COL3A1  ALDH1A2  CNTNAP2  CRABP1  SVEP1  LAMA2  BMP5  DCC  PALMD  F13A1  SLIT2  KCNA1  EDNRA  CDH11  KLHL41  HMCN1  PCDH18  SFRP2  SGCD  GPC3  NID2  MMP2  SAMD11  FGFR2  TMEM119  RAB3C  CCDC3  CTHRC1  EPHA3  ABCA13 | **11**  ID4  EREG  PCSK1  CCDC81  ITGA8  CXCL8  CDKN2C  DKK1  NOTCH4  ADRB1  EMCN | **46**  SAMD11  ID4  LINC02593  EMCN  ACTC1  ST8SIA6  ART3  SMAD9  LGR5  EBF3  ID2  TMEM26  NPY1R  ITGA8  PCSK1  EREG  NOG  RIPOR2  ADGRB3  IGFBPL1  UNC5C  ID1  SLC7A14  EXOC3L2  ELFN2  MGAT4C  LRRN3  LUZP2  TRIL  UNC5B  DLX2  LIMCH1  SPOCK1  ST6GALNAC3  FGFR3  MLIP  NPNT  NEB  DKK1  HAS2  CDKN2C  STC1  ADAMTS16  TBX2  BMP2  CHRM2  TRPC3 | **22**  SAMD11  LINC02593  UNC5C  CDH9  MLIP  IL1RAPL2  ST8SIA6  ART3  LRRC7  ADAMTS16  TRIL  EBF3  NPY1R  NPNT  ACTC1  FREM2  RPL11P3  IRS4  SLIT2-IT1  FIBIN  LINC02024  TMEM26 |
| Down-regulated | **6**  FGF5  NPPB  SRGN  TRIM58  KRT7  CLDN1 | **3**  CLDN1  SRGN  KRT7 | **10**  OXTR  CDH5  MT1L  RELN  TRIM58  NTN4  FGF5  SRGN  CLDN1  KRT7 | **31**  SAA1  MAD2L1P1  FAT2  LRRC7  CH25H  POLR2J3  HSPE1P13  TAS2R46  PCDHGA12  MFAP4  SPTBN5  DCDC2  TAS2R31  ARMH1  SLC30A10  TAS2R19  ABCA9  AACSP1  ACVR1C  APRG1  RPL11P3  SNORD62B  ASS1  LINC01422  RIPOR3  BMF  POLR2J2  BCL2L11  DRD2  LINC00278  RNY4P34 | **10**  SPTBN5  ASS1  IQSEC3  DCDC2  ACVR1C  SLC13A4  TMEM40  BCL2L11  SLC30A10  BMF | **2**  CLDN1  NTN4 |

**Supplementary Table 3. List of selected top transcripts selectively changed as a result of the culture of MB-DMD on the indicated hydrogel types.** Changes were indicated by NGS analysis compared to cells cultured on collagen I.

| **Comparison** | **R1 vs COL I** | **R1-SDF1-X vs COL I** | **R4-IL4-Y vs COL I** |
| --- | --- | --- | --- |
| Up-regulated | **5**  ASS1  IFITM1  LINC00278  LUM  SMOC2 | **0** | **28**  ABCA13 ADAMTS16 ALDH1A2 ART3 BMP5  CCDC3  CDH11  CNTNAP2  CTHRC1  DCC  EDNRA  EPHA3  F13A1  FIBIN  GPC3  KLHL41  LAMA2  LRRN3  MMP2  PALMD  PCDH18  RAB3C  SAMD11  SFRP2  SGCD  SLIT2  ST8SIA6  TRIL |

**Supplementary Table 4. List of selected top transcripts selectively changed as a result of the culture of MB-DMD on the indicated hydrogel types.** Changes were indicated by NGS analysis compared to cells cultured on R1 hydrogel.

| **Comparison** | **R1-SDF1-X vs R1** | **R4-IL4-Y vs R1** |
| --- | --- | --- |
| Up-regulated | **4**  ADRB1  CCDC81  CXCL8  NOTCH4 | **40**  ACTC1  ADAMTS16  ADGRB3  ART3  BMP2  CHRM2  DLX2  EBF3  ELFN2  EXOC3L2  FGFR3  HAS2  ID1  ID2  IGFBPL1  LGR5  LIMCH1  LINC02593  LRRN3  LUZP2  MGAT4C  MLIP  NEB  NOG  NPNT  NPY1R  RIPOR2  SAMD11  SLC7A14  SMAD9  SPOCK1  ST6GALNAC3  ST8SIA6  STC1  TBX2  TMEM26  TRIL  TRPC3  UNC5B  UNC5C |

**Supplementary Table 5. List of selected top transcripts, changed as a result of MB-DMD culture on R4-IL4-Y hydrogel.** Changes were indicated by NGS analysis compared to cells cultured in at least two other experiment variants, i.e. on collagen I, R1 and/or R1-SDF1-X hydrogel.

| **Comparison** | **R4-IL4-Y vs COL I** | **R4-IL4-Y vs R1** | **R4-IL4-Y vs R1-SDF1-X** |
| --- | --- | --- | --- |
| Up-regulated | **28**  ABCA13 **ADAMTS16** ALDH1A2 **ART3** BMP5  CCDC3  CDH11  CNTNAP2  CTHRC1  DCC  EDNRA  EPHA3  F13A1  **FIBIN**  GPC3  KLHL41  LAMA2  **LRRN3**  MMP2  PALMD  PCDH18  RAB3C  **SAMD11**  SFRP2  SGCD  SLIT2  **ST8SIA6**  **TRIL** | **40**  **ACTC1**  **ADAMTS16**  ADGRB3  **ART3**  BMP2  CHRM2  DLX2  **EBF3**  ELFN2  EXOC3L2  FGFR3  HAS2  ID1  ID2  IGFBPL1  LGR5  LIMCH1  **LINC02593**  **LRRN3**  LUZP2  MGAT4C  **MLIP**  NEB  NOG  **NPNT**  **NPY1R**  RIPOR2  **SAMD11**  SLC7A14  SMAD9  SPOCK1  ST6GALNAC3  **ST8SIA6**  STC1  TBX2  **TMEM26**  **TRIL**  TRPC3  UNC5B  **UNC5C** | **22**  **ACTC1**  **ADAMTS16**  **ART3**  CDH9  **EBF3**  **FIBIN**  FREM2  IL1RAPL2  IRS4  LINC02024  **LINC02593**  LRRC7  **MLIP**  **NPNT**  **NPY1R**  RPL11P3  **SAMD11**  SLIT2-IT1  **ST8SIA6**  **TMEM26** **TRIL**  **UNC5C** |

**Supplementary Table 6. List of top transcripts changed as a result of culture of MB-DMD on different hydrogels and selected for RT-qPCR analysis.** Changes were indicated by NGS analysis between indicated samples.

| **Gene name** | **Role** | **Notes** |
| --- | --- | --- |
| ACTC1 | Actin, alpha cardiac muscle 1, intermediate form. Actins are highly conserved proteins that are involved in various types of cell motility and are ubiquitously expressed in all eukaryotic cells. | Elevated in cells cultured on R4-IL4-Y vs other conditions (COL I, R1 and/or R1-SDF-1-X) |
| ADAMTS16 | ADAM metallopeptidase with thrombospondin type 1 motif 16. | Elevated in cells cultured on R4-IL4-Y vs other conditions (COL I, R1 and/or R1-SDF-1-X**)** |
| BMP2 | Bone morphogenetic protein 2. Induces cartilage and bone formation. Stimulates the differentiation of myoblasts into osteoblasts via the EIF2AK3-EIF2A-ATF4 pathway. Belongs to the TGF-beta family. | Elevated in cells cultured on R4-IL4-Y vs R1 |
| CDH9 | Cadherin-9. Cadherins are calcium-dependent cell adhesion proteins. They preferentially interact with themselves in a homophilic manner in connecting cells; thus contributing to the sorting of heterogeneous cell types. | Elevated in cells cultured on R4-IL4-Y vs R1-SDF-1-X |
| CXCL8 | Interleukin-8. IL-8 is a chemotactic factor that attracts neutrophils, basophils, and T-cells, but not monocytes. | Elevated in cells cultured on R1-SDF-1-X vs R1 |
| FIBIN | Fin bud initiation factor homolog. Engaged in Wnt and retinoid acid signalling pathways. | Elevated in cells cultured on R4-IL4-Y vs other conditions (COL I, R1 and/or R1-SDF-1-X) |
| FREM2 | FRAS1-related extracellular matrix protein 2. Extracellular matrix protein required for maintenance of the integrity of the epithelium and adhesion. Belongs to the FRAS1 family. | Elevated in cells cultured on R4-IL4-Y vs R1-SDF-1-X |
| ID1 | DNA-binding protein inhibitor ID-1. Plays a role in cell growth, senescence, and differentiation.  Negatively regulates the basic helix-loop-helix (bHLH) transcription factors. | Elevated in cells cultured on R4-IL4-Y vs R1 |
| ID2 | DNA-binding protein inhibitor ID-2. Transcriptional regulator which negatively regulates the basic helix-loop-helix (bHLH) transcription factors by forming heterodimers and inhibiting their DNA binding and transcriptional activity. Implicated in regulating a variety of cellular processes, including cellular growth, senescence, differentiation, apoptosis, and angiogenesis. Inhibits skeletal muscle and cardiac myocyte differentiation. | Elevated in cells cultured on R4-IL4-Y vs R1 |
| IRS4 | Insulin receptor substrate 4. Acts as an interface between multiple growth factor receptors possessing tyrosine kinase activity, such as insulin receptor, IGF1R and FGFR1, and a complex network of intracellular signalling molecules containing SH2 domains. Involved in the IGF1R mitogenic signalling pathway. | Elevated in cells cultured on R4-IL4-Y vs R1-SDF-1-X |
| LGR5 | Leucine-rich repeat-containing G-protein coupled receptor 5. Receptor for R-spondins that potentiates the canonical Wnt signalling pathway. Upon binding to R-spondins (RSPO1, RSPO2, RSPO3 or RSPO4), associates with phosphorylated LRP6 and frizzled receptors that are activated by extracellular Wnt receptors, triggering the canonical Wnt signalling pathway to increase expression of target genes. Expressed in myogenic progenitors. | Elevated in cells cultured on R4-IL4-Y vs R1 |
| MLIP | Muscular LMNA interacting protein. Transcription factor responsible for localization of cell nuclei in skeletal muscles. | Elevated in cells cultured on R4-IL4-Y vs other conditions (COL I, R1 and/or R1-SDF-1-X) |
| NEB | Nebulin. This giant muscle protein may be involved in maintaining the structural integrity of sarcomeres and the membrane system associated with the myofibrils. Binds and stabilize F-actin. | Elevated in cells cultured on R4-IL4-Y vs R1 |
| NOG | Noggin. Inhibitor of bone morphogenetic proteins (BMP) signalling. Stimulates myogenic differentiation of satellite cells. Inhibits chondrocyte differentiation through its interaction with GDF5 and, probably, GDF6. | Elevated in cells cultured on R4-IL4-Y vs R1 |
| NOTCH4 | Neurogenic locus notch homolog protein 4. Functions as a receptor for membrane-bound ligands Jagged1, Jagged2 and Delta1 to regulate cell-fate determination. Upon ligand activation through the released notch intracellular domain (NICD) it forms a transcriptional activator complex with RBPJ/RBPSUH and activates genes of the enhancer of split locus. Affects the implementation of differentiation, proliferation and apoptotic programs. | Elevated in cells cultured on R1-SDF-1-X vs R1 |
| SMAD9 | Mothers against decapentaplegic homolog 9. Transcriptional modulator activated by BMP (bone morphogenetic proteins) type 1 receptor kinase. | Elevated in cells cultured on R4-IL4-Y vs R1 |
| TBX2 | T-box transcription factor TBX2. Involved in the transcriptional regulation of genes required for mesoderm differentiation. Probably plays a role in limb pattern formation. | Elevated in cells cultured on R4-IL4-Y vs R1 |

**Supplementary Table 7. STRING analysis of top 17 transcripts changed as a result of culture of MB-DMD on different hydrogels.** Changes were indicated by NGS analysis between indicated samples and next verified by RT-qPCR analysis. Neighborhood_on_chromosome and gene_fusion columns were skipped as no hits were found. For phylogenetic_cooccurrence and homology the only hits were found for ID1 and ID2 with 0.066 and 0.881 values, respectively.

| **#node1** | **node2** | **node1_ string_id** | **node2_ string_id** | **co- expression** | **experimentally**  **determined_**  **interaction** | **database_**  **annotated** | **automated_**  **text mining** | **combined_**  **score** |
| --- | --- | --- | --- | --- | --- | --- | --- | --- |
| BMP2 | TBX2 | 9606.ENSP00000368104 | 9606.ENSP00000240328 | 0.050 | 0.071 | 0 | 0.611 | 0.626 |
| BMP2 | LGR5 | 9606.ENSP00000368104 | 9606.ENSP00000266674 | 0.067 | 0 | 0 | 0.530 | 0.543 |
| BMP2 | CXCL8 | 9606.ENSP00000368104 | 9606.ENSP00000306512 | 0.067 | 0 | 0 | 0.419 | 0.434 |
| BMP2 | NOG | 9606.ENSP00000368104 | 9606.ENSP00000328181 | 0.042 | 0.937 | 0.900 | 0.996 | 0.999 |
| BMP2 | ID1 | 9606.ENSP00000368104 | 9606.ENSP00000365280 | 0.086 | 0 | 0 | 0.586 | 0.605 |
| BMP2 | SMAD9 | 9606.ENSP00000368104 | 9606.ENSP00000369154 | 0.044 | 0.161 | 0.500 | 0.719 | 0.872 |
| CXCL8 | BMP2 | 9606.ENSP00000306512 | 9606.ENSP00000368104 | 0.067 | 0 | 0 | 0.419 | 0.434 |
| CXCL8 | NOG | 9606.ENSP00000306512 | 9606.ENSP00000328181 | 0 | 0 | 0 | 0.484 | 0.483 |
| ID1 | ID2 | 9606.ENSP00000365280 | 9606.ENSP00000234091 | 0.115 | 0 | 0 | 0.462 | 0.516 |
| ID1 | NOG | 9606.ENSP00000365280 | 9606.ENSP00000328181 | 0.048 | 0 | 0 | 0.445 | 0.449 |
| ID1 | SMAD9 | 9606.ENSP00000365280 | 9606.ENSP00000369154 | 0.055 | 0 | 0 | 0.494 | 0.501 |
| ID1 | BMP2 | 9606.ENSP00000365280 | 9606.ENSP00000368104 | 0.086 | 0 | 0 | 0.586 | 0.605 |
| ID2 | NOG | 9606.ENSP00000234091 | 9606.ENSP00000328181 | 0 | 0 | 0 | 0.426 | 0.426 |
| ID2 | TBX2 | 9606.ENSP00000234091 | 9606.ENSP00000240328 | 0.060 | 0 | 0 | 0.480 | 0.491 |
| ID2 | ID1 | 9606.ENSP00000234091 | 9606.ENSP00000365280 | 0.115 | 0 | 0 | 0.462 | 0.516 |
| LGR5 | BMP2 | 9606.ENSP00000266674 | 9606.ENSP00000368104 | 0.067 | 0 | 0 | 0.530 | 0.543 |
| LGR5 | NOG | 9606.ENSP00000266674 | 9606.ENSP00000328181 | 0 | 0 | 0 | 0.773 | 0.773 |
| NOG | ID2 | 9606.ENSP00000328181 | 9606.ENSP00000234091 | 0 | 0 | 0 | 0.426 | 0.426 |
| NOG | TBX2 | 9606.ENSP00000328181 | 9606.ENSP00000240328 | 0.042 | 0 | 0 | 0.453 | 0.453 |
| NOG | LGR5 | 9606.ENSP00000328181 | 9606.ENSP00000266674 | 0 | 0 | 0 | 0.773 | 0.773 |
| NOG | CXCL8 | 9606.ENSP00000328181 | 9606.ENSP00000306512 | 0 | 0 | 0 | 0.484 | 0.483 |
| NOG | ID1 | 9606.ENSP00000328181 | 9606.ENSP00000365280 | 0.048 | 0 | 0 | 0.445 | 0.449 |
| NOG | SMAD9 | 9606.ENSP00000328181 | 9606.ENSP00000369154 | 0 | 0 | 0 | 0.495 | 0.495 |
| NOG | BMP2 | 9606.ENSP00000328181 | 9606.ENSP00000368104 | 0.042 | 0.937 | 0.900 | 0.996 | 0.999 |
| SMAD9 | NOG | 9606.ENSP00000369154 | 9606.ENSP00000328181 | 0 | 0 | 0 | 0.495 | 0.495 |
| SMAD9 | ID1 | 9606.ENSP00000369154 | 9606.ENSP00000365280 | 0.055 | 0 | 0 | 0.494 | 0.501 |
| SMAD9 | BMP2 | 9606.ENSP00000369154 | 9606.ENSP00000368104 | 0.044 | 0.161 | 0.500 | 0.719 | 0.872 |
| TBX2 | ID2 | 9606.ENSP00000240328 | 9606.ENSP00000234091 | 0.060 | 0 | 0 | 0.480 | 0.491 |
| TBX2 | NOG | 9606.ENSP00000240328 | 9606.ENSP00000328181 | 0.042 | 0 | 0 | 0.453 | 0.453 |
| TBX2 | BMP2 | 9606.ENSP00000240328 | 9606.ENSP00000368104 | 0.050 | 0.071 | 0 | 0.611 | 0.626 |

**Supplementary Figure 1**

 **Supplementary Figure 2**

**Supplementary Figure 3**

**Supplementary Figure 4**

**Supplementary Figure 5**

**Supplementary Figure 6**

A

hiPSC-DMD colI

hiPSC-DMD R1

hiPSC-DMD R1-RGD

hiPSC-DMD R1-SDF1-X

hiPSC-DMD R4-RGD

hiPSC-DMD R4-IL4-Y

B

hiPSC-LMNβ1 colI

hiPSC-LMNβ1 R1

hiPSC-LMNβ1 R1-RGD

hiPSC-LMNβ1 R1-SDF1-X

hiPSC-LMNβ1 R4-RGD

hiPSC-LMNβ1 R4-IL4-Y

**Supplementary Figure 7**
